# The Role of Benzylpenicilloyl Epimers in Specific IgE Recognition

**DOI:** 10.3389/fphar.2021.585890

**Published:** 2021-02-26

**Authors:** Cristobalina Mayorga, Maria I. Montañez, Francisco Najera, Gador Bogas, Tahía D. Fernandez, David Rodríguez Gil, Ricardo Palacios, Maria J. Torres, Yolanda Vida, Ezequiel Perez-Inestrosa

**Affiliations:** ^1^ Allergy Research Group, Instituto de Investigación Biomédica de Málaga-IBIMA, Málaga, Spain; ^2^ Allergy Unit, Hospital Regional Universitario de Málaga, Málaga, Spain; ^3^ Centro Andaluz de Nanomedicina y Biotecnología-BIONAND, Parque Tecnológico de Andalucía, Málaga, Spain; ^4^ Universidad de Málaga-IBIMA Departamento de Química Orgánica, Málaga, Spain; ^5^ Universidad de Málaga-IBIMA, Departamento de Biología celular, Genética y Fisiología, Málaga, Spain; ^6^ Diater Laboratorios S.A., Madrid, Spain; ^7^ Universidad de Málaga-IBIMA, Departamento de Medicina, Málaga, Spain

**Keywords:** antigenic determinant, diagnostic test, drug allergy, penicillin, specific IgE

## Abstract

The high prevalence of allergy to β-lactam antibiotics is a worldwide issue. Accuracy of diagnostic methods is important to prove tolerance or allergy, with skin test considered the best validated *in vivo* method for diagnosing immediate reactions to β-lactams. Although drug provocation test is the reference standard, it cannot be performed in highly risk reactions or in those with positive skin tests. For skin tests, the inclusion of major and minor determinants of benzylpenicillin (BP) is recommended. Commercial skin test reagents have changed along time, including as minor determinants benzylpenicillin, benzylpenicilloate (BPO), and benzylpenilloate (PO). Major determinants consists of multivalent conjugates of benzylpenicilloyl coupled through amide bond to a carrier polymer, such as penicilloyl-polylysine (PPL) or benzylpenicilloyl-octalysine (BP-OL). The chemical stability of such reagents has influenced the evolution of the composition of the commercial kits, as this requirement is necessary for improving the quality and standardization of the product. In this work, we provide a detailed study of the chemical stability of BP determinants. We observed that those structures suffer from an epimerization process in C-5 at different rates. Butylamine-Benzylpenicilloyl conjugates (5*R*,6*R*)-Bu-BPO and (5*S*,6*R*)-Bu-BPO were selected as a simple model for mayor determinant to evaluate the role of the different epimers in the immunoreactivity with sera from penicillin-allergic patients. *In vitro* immunoassays indicate that any change in the chemical structure of the antigenic determinant of BP significantly affects IgE recognition. The inclusion of stereochemically pure compounds or mixtures may have important implications for both the reproducibility and sensitivity of *in vivo* and *in vitro* diagnostic tests.

## Introduction

β-lactam antibiotics (BLs) family is nowadays the first choice for the treatment of a large number of bacterial infections. Its extended use probably explains why they are the drugs most frequently involved in drug hypersensitivity reactions, which have important consequences in terms of safety, durability and effectiveness of treatment. Alternative antibiotics may be less effective, more toxic and expensive, and lead to increased bacterial resistance (Doña et al., 2017). Therefore, a correct diagnosis of drug allergy is very important for an adequate prescription of the drug in order to avoid risks for the patient (Montañez et al., 2015). The first approach for evaluating the patients is a detailed clinical history, which is often very difficult to obtain. After that, the initial choice is frequently skin test (Doña et al., 2019), and *in vitro* tests which are mainly recommended in patients with dermatological problems, for whom skin test can produce equivocal results, or in high risk patients to reduce the risk of systemic reactions (Mayorga et al., 2016a; Doña et al., 2017). However, *in vitro* tests are less sensitive than skin test to diagnose penicillin allergy. Although many factors could be involved, one of the most important ones could be the drug or drug metabolite included in the test, in which IgE from patients shows specific recognition (Torres et al., 2010; Mayorga et al., 2016a). Because of patient safety, above described methods are the preferred for diagnosis of drug allergy (Demoly et al., 2014; Doña et al., 2019). However if skin test and *in vitro* tests are negative, drug provocation tests, also called oral challenge, is the reference standard test required to confirm diagnosis (Torres et al., 2017; Demoly et al., 2014).

The most representative drug model nowadays in the study of immediate (or IgE-mediated) reactions to BLs continues to be Benzylpenicillin (BP) (Levine and Ovary 1961; Batchelor et al., 1965). The main reason is its well-known reactivity based on the nucleophilic attack of free amino groups of proteins to the extremely reactive β-lactam ring. The opening of the high strain four member ring is an efficient process that leads to the formation of the benzylpenicilloyl determinant. The benzylpenicilloyl amide linked to protein constitutes the reaction product of the 95% of the penicillin molecules that reacts with proteins under physiological conditions, and it is thus considered the major antigenic determinant of BP. The remaining BP molecules react in a different way, resulting in other structures considered as minor determinants, such as BPO acid form and benzylpenilloic acid (PO) (Martin-Serrano et al., 2016). Many of these structures can be recognized in a different way by IgE from allergic patients, and therefore they are used with diagnostic purposes in *in vivo* and *in vitro* tests.

**FIGURE 1:**
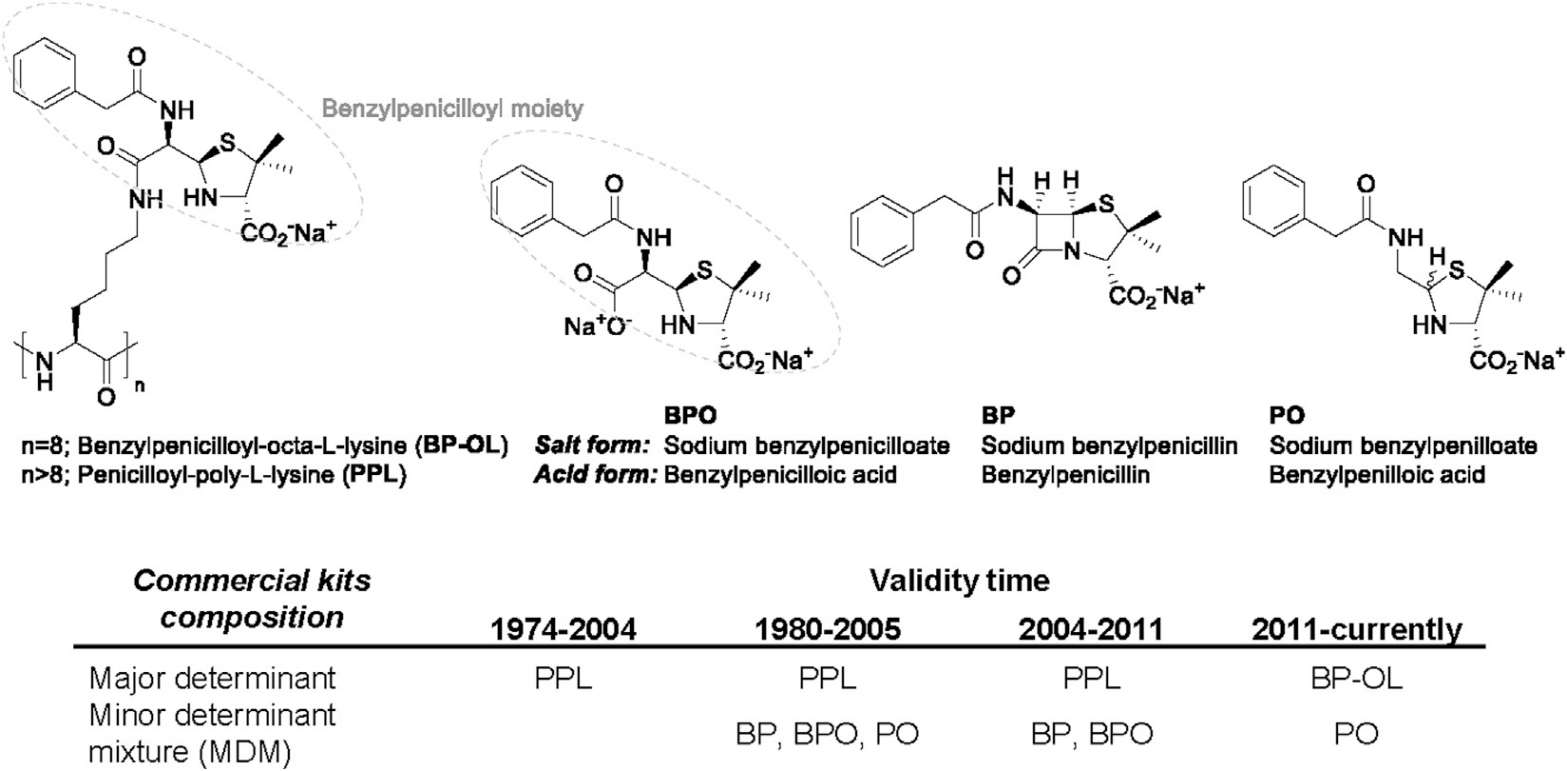
Mayor (BP-OL and PPL) and minor (BPO, BP and PO) determinants of BP traditionally used in skin tests. Evolution of the commercial kits composition over the time.

In the case of skin testing, BPO acid, BPO amide forms and PO have been used, showing in some cases higher sensitivity than when using BP itself, probably because the two forms are able to bind better the polyclonal IgE. Extended studies have been carried out since 1977, when the suitability of the major determinant and the different minor determinants was evaluated. In fact, commercial kits have continuously changed over the time. The first commercial kit, in 1974, contained only penicilloyl-poly-L-lysine (PPL) as major determinant. In 1980s, commercial kit included the traditional BP reagents: PPL and a minor determinant mixture (MDM), containing BP, BPO and PO, Figure 1. Such kits were removed from the market between 2004 and 2005 (Ariza et al., 2015). In 2004 a different composition mixture was commercialized, including PPL as major determinant, and only BP and BPO as MDM. In 2011, this formulation was substituted by the more stable benzylpenicilloyl-octa-L-lysine (BP-OL) as major determinant and PO as minor determinant (Fernández et al., 2013; Fernandez et al., 2017). Indeed the diagnosis guidelines from Europe (Maria J. Torres et al., 2003) and North America (Joint Task Force on Practice Parameters et al., 2010) recommended the use of these antigenic determinants of penicillin in skin testing. Nowadays, the commercial determinants in skin test reagents used are BPO-lysine polymer conjugates (BP-OL or PPL, in Europe and United States respectively), together with PO (only in Europe) (Ariza et al., 2015; Martin-Serrano et al., 2016). However, it should be noted that the commercial determinants available depend on the country.

In the case of *in vitro* tests, immunoassays, which are based on the determination of specific IgE, have been the most widely employed technique. The amide form of BPO covalently bound to PLL is included in solid phase of both commercial and homemade immunoassays (Montañez et al., 2011a). In fact, the ImmunoCAP tests available for several penicillins (BP, amoxicillin, ampicillin and penicillin V) is based on the penicilloyl-PLL conjugate attached to cellulose solid phase (Fontaine et al., 2007). The homemade RAST (Radio Allergo Sorbent Test) also employs BPO-PLL conjugates attached to a solid phase which, in this case, is a cellulose paper disc. The main reason for the endorse use of such compounds in diagnosis is their high specific recognition by sIgE from penicillin-allergic patients, together with the straightforward reaction of BP with amine nucleophiles and the stability of the penicilloyl determinants formed. This has allowed modifications in homemade RAST assays, in which different carriers and solid phases have been successfully tested (Montañez et al., 2008; Ruiz-Sanchez et al., 2012; Vida et al., 2013; Mayorga et al., 2016b). However, the IgE recognition with BP determinants different from BPO amide structure has not been studied in detail.

Immunoassays are a valuable tool for evaluating the IgE recognition of the different structures, and in this study we have used them to address the immunological recognition of antigenic determinants and very related chemical structures derived from their storage conditions. In fact, the chemical stability of BP skin test reagents has influenced the evolution of the composition of the commercial kits, as this requirement is necessary for improving the quality and standardization of the product.

Experimental Nuclear Magnetic Resonance (NMR) studies and theorical calculations are suitable methods to get insight into the stability of the determinants and the structural mechanisms concerned. Herein, we report a detailed study of the chemical stability of the BP determinants traditionally used in skin tests, elucidating the chemical process involved, the resulting isomers formed as well as their structural immunoreactivity (or immunological recognition).

## Materials and Methods

Standard chemicals were obtained from Aldrich or VWR and used without further purification. Phosphate buffer saline (PBS, pH∼7.4) was prepared as described elsewhere (Blanca et al., 1992), by dissolving 40 mg of NaCl, 1 mg of KH_2_PO_4_, 4.5 mg of Na_2_HPO_4_ and 1 mg of KCl in 5 ml of either H_2_O or D_2_O (99.96% D, from VWR). Carbonate buffer (pH∼10.2) was prepared by disolving 14.5 mg of Na_2_CO_3_ and 9.5 mg of NaHCO_3_ in 5 ml of either H_2_O or D_2_O. BP sodium salt, sodium benzylpenicilloate and sodium benzylpenilloate were supplied by DIATER, SA. Benzylpenicilloyl-Butylamine (Bu-BPO) was prepared as previously described (Sánchez-Sancho et al., 2002). NMR samples were prepared by dissolving 8 mg of the corresponding determinant (BP, BPO, PO or Bu-BPO) in 0.75 ml of the corresponding deuterated solvent and solutions were kept at the indicated temperature until the spectra were recorded. ^1^H-NMR spectra were measured in the indicated deuterated solvent on a Bruker Ascend 400 MHz spectrometer. Proton chemical shifts (δ) are reported with the solvent resonance employed as the internal standard (D_2_O δ 4.79).

### Computational Studies

All calculations were performed with the Gaussian 16 package (Frisch et al., 2016). In all simulations, the solvent effect was considered including the polarizable continuum model (PCM) (Tomasi et al., 2005), and water as solvent. The potential energy scans were done for compounds BPO, PO and Bu-BPO at DFT/B3LYP/6-311G (2 days,p) level of theory using the dihedral angle (H_6_-C_6_-C_5_-H_5_) as variable. The minima obtained in the potential energy scan was used as the initial point for the optimization of their structures at B3LYP/6-311++G (2 days,p) level or theory. The absence of a negative frequency in analytical Hessian calculations confirmed that all the geometries found were minima. To study the intramolecular hydrogens bonds in these molecules, a topological analysis was done using the Bader’s atoms in molecules (AIM) theory (Bader 1991), and the Multiwfn 3.6 program (Lu and Chen 2012; Lu 2020).

### Selection of Patients

Patients with a clinical history of an immediate allergic reaction to BP diagnosed following European Academy of Allergy and Clinical Immunology (EAACI) and European Network of Drug Allergy (ENDA) guidelines (Doña et al., 2019; Romano et al., 2020). The studied group was obtained from the Regional University Hospital of Málaga Drug Allergy database, from which we selected eleven cases with a positive skin test and *in vitro* detection of sIgE to the BP greater than 4.5%, measured by direct RAST. Data from the patients included in the study are displayed in Supplementary Table S1 (ESI).

The study was approved by the institutional review board, and informed consent for all procedures was obtained from all patients.

### Skin Test

Skin prick tests and, if negative, intradermal tests were performed as described (Doña et al., 2019; Romano et al., 2020), using PPL (DAP, Diater, Leganés, Spain) at 1.07·10^−2^ M, minor determinant mixture (MDM: BP, BPO and PO) at 1.5 M. Since May 2011 DAP composition has changed and includes the major determinant BP-OL at 0.04 mg/ml, equivalent to 8.64·10^−5^ M concentration of the benzylpenicilloyl moiety, and the minor determinant (MD) at 0.5 mg/ml, equivalent to 1.5·10^−3^ M concentration of PO.

Readings were done after 20 min and considered positive: 1) In skin prick test, if a wheal larger than 3 mm surrounded by erythema appeared, with a negative response to the control saline; 2) In intradermal tests, if the increase in diameter of the wheal area marked initially was greater than 3 mm surrounded by erythema. Positive data expressed as two diameters being one of them the straight line connecting the two most distant points of the wheal and the other the one at 180° (Brockow et al., 2002).

### *In vitro* Specific IgE Determination

RAST was done using BP conjugated to PLL functionalized-cellulose discs resulting in BPO-PLL in the solid phase, as described (Antunez et al., 2006; Ariza et al., 2016), and radiolabeled anti-IgE antibody (kindly provided by Thermo Fisher Scientific and radiolabeled in our laboratory) (Martín-Serrano et al., 2020). Results were expressed as percentage from a maximum counts and samples were considered positive if the percentage was higher than 2.5% of label uptake, which was the mean + 2SD of a negative control group.

### Competitive Inhibition Immunoassay

Solution of inhibitors were prepared as follows. Inhibitor 1: (*5R,6R*)-Bu-BPO was freshly dissolved in PBS; Inhibitor 3: (*5R,6R*)-Bu-BPO in carbonate buffer for 7 days afforded the mixture including 55% of its epimer, (*5S,6R*)-Bu-BPO; Inhibitor 2: a mixture with equivalent volumes of previous samples (inhibitors 1 and 3) resulted in the mixture containing 22.5% of C-5 epimer. RAST inhibition assay was done as described (Antunez et al., 2006; Ariza et al., 2016), incubating sera from patients and the Bu-BPO determinants (as inhibitors) in three ten-fold decreasing concentrations (100 mM–1 mM) for 18 h at room temperature. After this, the BPO-PLL discs were added, and RAST procedure was performed as described above. The results were expressed as percentage inhibition with respect to the serum incubated only with PBS (non-inhibited serum). Comparison of the inhibition capacity of the different inhibitors was performed at 50% inhibition using the IC50 and statistically analyzed for differences among the distributions of the three inhibitors by Friedman test, Wilcoxon test was used to make comparisons between two pair groups. All assays were performed at room temperature.

## Results

### Stability of the Minor Determinants

NMR Studies were performed to evaluate the stability of the minor determinant reagents used along the last years in solution, in a 7–12 pH range. Solutions of (3*S*,5*R*,6*R*)-BP resulted in one pure product. In the ^1^H-NMR it can be clearly distinguished the signal corresponding to H-5, H-6 and H-3, bonded to the β–lactam moiety (Busson and Vanderhaeghe 1976). The compound was completely stable for several days, in both D_2_O and physiological conditions (PBS, pH∼7.4) (Supplementary Figure S1, ESI).

The obtainment of the BPO determinant from BP is a well described procedure (Munro et al., 1978). The formation of BPO is a spontaneous and efficient process when dissolving BP in aqueous basic media (Pajares et al., 2020). Such reaction could be easily followed by ^1^H-NMR spectroscopy, resulting in a unique compound with a well defined stereochemistry derived from the original BP (Supplementary Figure S2, ESI). (3*S*,5*R*,6*R*)-benzylpenicilloate can be easily distinguished from BP by NMR. Signals corresponding to H-5 and H-6 suffer a displacement toward high fields and appear more separated from each other when the β-lactam ring is opened. Additionally, signal corresponding to H-3 displaces from 4.20 to 3.91 ppm. The separation between the signal of both methyl groups of the thiazolidine ring, namely CH_3_(α) and CH_3_(β), increased too.

**FIGURE 2:**
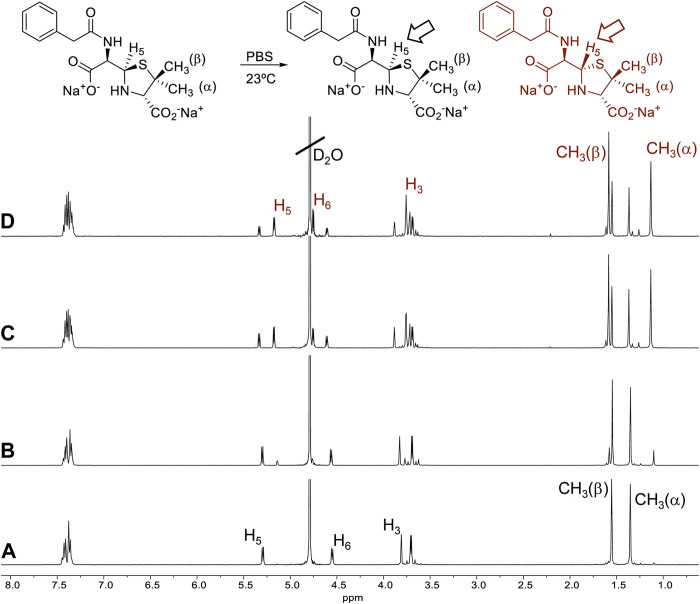
^1^H-NMR spectra of a solution of BPO in PBS/D_2_O, pD∼7.4 at 23°C, **(A)** freshly prepared and after **(B)** 3 h, **(C)** 24 h and **(D)** 48 h.

To evaluate the stability of BPO aqueous solution at different conditions, ^1^H-NMR spectra were recorded over time. We observed important changes, indicating the formation of a new product. Signal displacements seem to indicate the formation of a diastereoisomer, in particular the epimer (3*S*,5*S*,6*R*)-benzylpenicilloate (Ghebre-Sellassie et al., 1984; Haginaka and Wakai 1985). To get more insight into the C-5 epimerization process, the stability of (3*S*,5*R*,6*R*)-BPO has been tested in aqueous media at different pH and temperatures. First, a solution of the compound in PBS/D_2_O (pH∼7.4) at room temperature was monitored over time (Figure 2).

**FIGURE 3:**
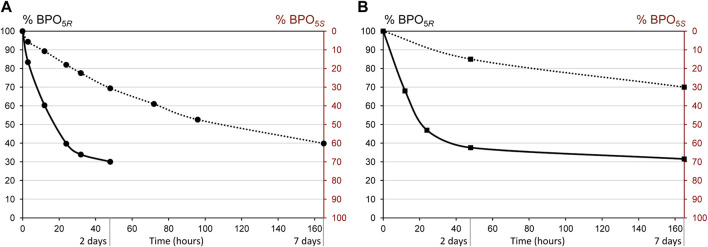
Stability of BPO in **(A)** PBS/D_2_O, pD∼7.4 and **(B)** D_2_O, pD = 6, at 23°C (solid line) and 4°C (dotted line).

Appearance of new signals was appreciable after 3 h (Figure 2B). The gap between the chemical shifts of the signals corresponding to the geminal methyl groups increased. Signal corresponding to H-5 displaced to higher field, whereas signal corresponding to H-6 displaced to lower field (from 4.55 to 4.75 ppm). The coupling constant between H-6 and H-5 also decreased from 5.3 Hz to 3.8 Hz. All these data confirm the formation of the (5*S*,6*R*)-BPO epimer. The process evolves until it reaches an equilibrium state after ∼30 h, in which approximately 70% of the (5*S*,6*R*)-BPO epimer is formed, while a 30% of the original (5*R*,6*R*)-BPO isomers remains in solution (Figure 3). To evaluate the effect of pH in the process, a solution of (5*R*,6*R*)-BPO in D_2_O was prepared and its evolution was monitored. The appearance of the same signal can be clearly observed after 15 h (Supplementary Figure S3, ESI). However, the equilibrium state was not reached until seven days after. The influence of temperature in the process was evaluated. When a solution of (5*R*,6*R*)-BPO in PBS/D_2_O (pH∼7.4) was monitored cooling at 4°C, we observed that the process is even slower, taking 7 days to reach a conversion of the 60% (Figure 3). The same effect was observed when monitoring the process in D_2_O at 4°C.

Similar studies were made to evaluate the stability in solution of commercial PO, which included (3*S*,5*R*)- and (3*S*,5*S*)-Benzylpenilloate diastereomers. Their solutions at physiological conditions were analyzed both at room (37°C) and low (4°C) temperature conditions. ^1^H-NMR spectra of the freshly prepared solutions show two products (Supplementary Figure S4, ESI), corresponding to 50:50 mixture of the PO diastereomers in C-5. The solution is stable, observing the same mixture (50:50 ratio) without any degradation product, after 48 h at room temperature, or 7 days at 4°C, at least. Similar behavior is observed when the solution is prepared using D_2_O as solvent (Supplementary Figure S5, ESI).

Table 1 shows the conversion rates for the epimerization of (5*R*,6*R*)-BPO and the equimolar mixture of (5*R*)-PO and (5*S*)-PO. Differences can be clearly observed between both compounds. For BPO, it is also noticeable the influence of the pH and temperature on the process, which is accelerated in basic media and high temperature.

**TABLE 1:** Conversion rates of the minor determinants epimerization process.

	PBS/D_2_O (pH∼7.4)		D_2_O (pH∼6)	
Time/T (°C)	23 ± 2°C	4 ± 1°C	23 ± 2°C	4 ± 1°C
% (5*R*,6*R*)-BPO remaining in solution				
0 h	100	100	100	100
24 h	40	82	47	
48 h	30	69	38	85
7 days		40	30	70
% (5*R*)-PO remaining in solution				
0 h	50	50	50	50
48 h	50		50	50
7 days		50		

### Stability of the Major Determinants

As previously exposed, the mayor determinants currently used in skin tests are BPO-lysine polymer conjugates (BP-OL or PPL). In order to simplify the study, a low molecular weight amine functionalized compound was used instead of amine functionalized polymers to generate the amide-BPO derivatives. We chose benzylpenicilloyl-butylamine (Bu-BPO) as model monomer compound. The reaction between butylamine and BP in aqueous media yielded the product corresponding to the aminolysis of the β-lactam ring (Bu-BPO) instantaneously (Sánchez-Sancho et al., 2002: Montañez et al., 2011b). The stability of Bu-BPO has been tested in similar conditions to those studied for minor determinants.

Solutions of Bu-BPO in aqueous media and PBS resulted perfectly stable at room temperature for 7 days (Supplementary Figures S6, S7, ESI), since signals corresponding to Bu-BPO in ^1^H-NMR spectra do not change in these conditions. To evaluate the stability of the determinant in basic media, solutions of Bu-BPO in carbonate buffer (pH∼10.2) at room temperature were evaluated. The formation of the (5*S*,6*R*)-Bu-BPO epimer can be observed in the NMR spectrum after 48 h (Supplementary Figure S8, ESI). The process evolves until approximately 55% of the (5*S*,6*R*)-Bu-BPO epimer is formed, while a 45% of the original (5*R*,6*R*)-Bu-BPO isomers remain in solution after 7 days (Table 2).

**TABLE 2:** Conversion rates of Bu-BPO epimerization process.

Time	D_2_O (pH∼6)	PBS/D_2_O (pH∼7.4)	Carbonate buffer/D_2_O (pH = 10.2)
% (5*R*,6*R*)-Bu-BPO remaining in solution			
0 h	100	100	100
48 h	100	100	70
7 days	100	100	45

### Immunological Studies

The ability of IgE in sera from BP-allergic patients to recognize BP determinants bearing different stereochemistry was studied by RAST inhibition. This assay consists in competitive serum IgE recognition between the solid phase (PLL-BPO conjugate attached to cellulose) and the different inhibitors (Bu-BPO 1) and its mixture with different percentage of its epimer at C-5 (2 (22.5%) and 3, (55%)) at different concentrations in the fluid phase.

BPO-Bu compound was selected because it presents the higher stability in aqueous solution at neutral pH, which permits controlling the precise chemical structures of inhibitors. Moreover, inhibitor 3, consisting of a mixture containing a 55% of its epimer in C-5 (45:55, *5R6R*:*5S6R*) obtained when reached equilibrium in basic aqueous media, was evaluated. In addition, inhibitor 2, a mixture containing midpoint of above concentrations of Bu-BPO and its epimer in C-5 (77.5:22.5, *5R6R*:*5S6R*) was also included.

**FIGURE 4:**
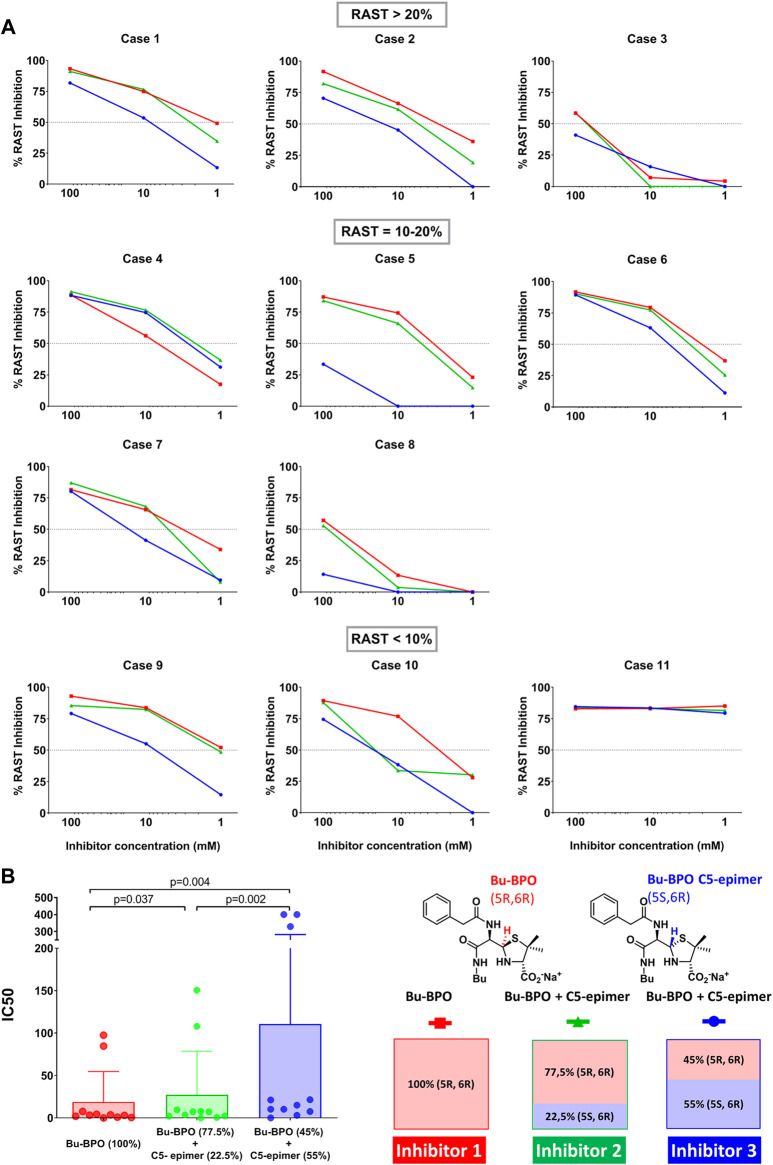
RAST inhibition assays. **(A)** Results obtained with eleven serum samples from patients allergic to penicillins with different BP-sIgE levels. The Bu-BPO and the mixtures including its epimer at C-5, at different proportion, were used as fluid phase inhibitors at three concentrations. Dots lines indicate 50% of inhibition in which sIgE recognition is considered. **(B)** IC50 for the three kinds of inhibitors (Bu-BPO and the two mixtures with its epimer in C-5) from the eleven sera included. Friedman test indicated statistically significant differences between the distributions of the three inhibitors (*p* = 0.0002). Wilcoxon test was used to make comparisons between two pair groups.

The immunological evaluation of Bu-BPO, and its mixtures with its epimer at C-5, by RAST inhibition (Figure 4A) showed, in most cases, a concentration dependent inhibition of BP-specific sera, with inhibitors following similar patterns in each serum independently of the BP-specific IgE levels, with the best recognition obtained with the inhibitor 1, with higher content of (*5R,6R*)-Bu-BPO.

*In vitro* IgE recognition is normally considered meaningful and positive when the inhibition percentage is higher than 50%. In general, at the maximum concentration of the determinants (100 mM) there was a positive inhibition of 100% of sera for inhibitors one and two and of 81.8% for the inhitor 3. This inhibition dropped at 1 mM concentration, being positive in three out of 11 cases (27%) for inhitor one and one out 11 cases (9%) for inhibitors 2 and 3, which contain the epimer.

In order to evaluate the role of the epimer in sIgE recognition from allergic patients, we calculated the half-maximal inhibitory concentration (IC50) for each inhibitor represented in Figure 4B. The median and interquartilic ranges of IC50 were 3.32 (IQ:0.96–7.7), 7.19 (IQ:2.14–9.57) and 15.11 (IQ: 7.64–329.3) for inhibitors 1, two and three respectively, indicanting a decrease of recognition with the increase of the epimer concentration. Statistical comparisons by Friedman test showed significant differences for the three inhibitors (*p* = 0.0002). The concentration to get 50% of inhibition was significantly lower for the inhibitor 1, which contains only (*5R,6R*)-Bu-BPO compared with the inhibitors 2 and 3, which contain 22.5 (*p* = 0.037) and 55% (*p* = 0.004) of C-5 epimer, respectively. Moreover, we found significant differences in the IC50 between the inhibitors 2 and 3, which contain 22.5 and 55% of the epimer, respectively (*p* = 0.002) (Figure 4B).

## Discussion

BP consumption has decreased over the three last decades, mainly substituted by amoxicillin (with or without clavulanic acid) and cephalosporins. However, skin test to BP determinants is still recomended in the BL allergy work up diagnosis (Doña et al., 2017). Different compositions have been used over time, including as minor determinants: BP (active principle), BPO (the hydrolysis product), and PO (the decarboxilated BPO); and as major determinants conjugates of lysine polymers: PPL and BP-OL. The change in compositions can be explained by stability issues (which must overcome quality and standardization requirements of the product in different countries) and diagnostic results. In fact, a recent study concluded that skin testing with BP can induce false-positive results in patients with a history of BL allergy, and that the addition of BP does not increase the skin test sensitivity obtained with classic BP determinants (Lacombe-Barrios et al., 2016). Moreover, false positive results issues have also been reported in commercial *in vitro* tests, ImmunoCAP, in patients with suspected IgE-mediated hypersensitivity to penicillins and a positive penicillin ImmunoCAP due to the presence of IgE antibodies to phenylethylamine. This is a common allergenic structure that shares structural fragments (benzyl group) of the penicillin determinants and inappropriately, is potentially present in the ImmunoCAP test (Johansson et al., 2013). In addition, experts from the United States have reported that ImmunoCAP to quantify sIgE to BP shows suboptimal sensitivity and low concordance with *in vivo* tests, probably because this immunoassay only identifies IgE to the major determinant (Macy et al., 2010).

Although BPO is no longer the most relevant hapten in immediate reactions to penicillins, major and minor determinants of BP continue to play a key role in drug allergy diagnosis (Fernández et al., 2013). These determinants consist of the precise chemical structures involved in the specific IgE molecular recognition. Therefore the stability of these molecules is an important factor for diagnosis evaluation, as a small change in the structure can affect the immunological recognition. Moreover, the stability of the determinants can also affect the reproducibility of the tests, which is a crucial point in diagnosis. In that sense, the stability of the determinants used has been evaluated in aqueous solutions in different pH conditions, ranging from pH 7 to 10.2. BP resulted stable enough in 6–7.2 pH range, however, by increasing basicity the β-lactam ring opens, resulting in the formation of BPO (Munro et al., 1978).

According to previous studies, BP degradation products, formed *in vitro* when the drug is longer in aqueous solution, are the sensitization agents rather than the BP molecule itself. Although authors did not identify these degradation products, we could assume that BPO and its epimer are involved (Neftel et al., 1984; Neftel et al., 1982).

**FIGURE 5:**
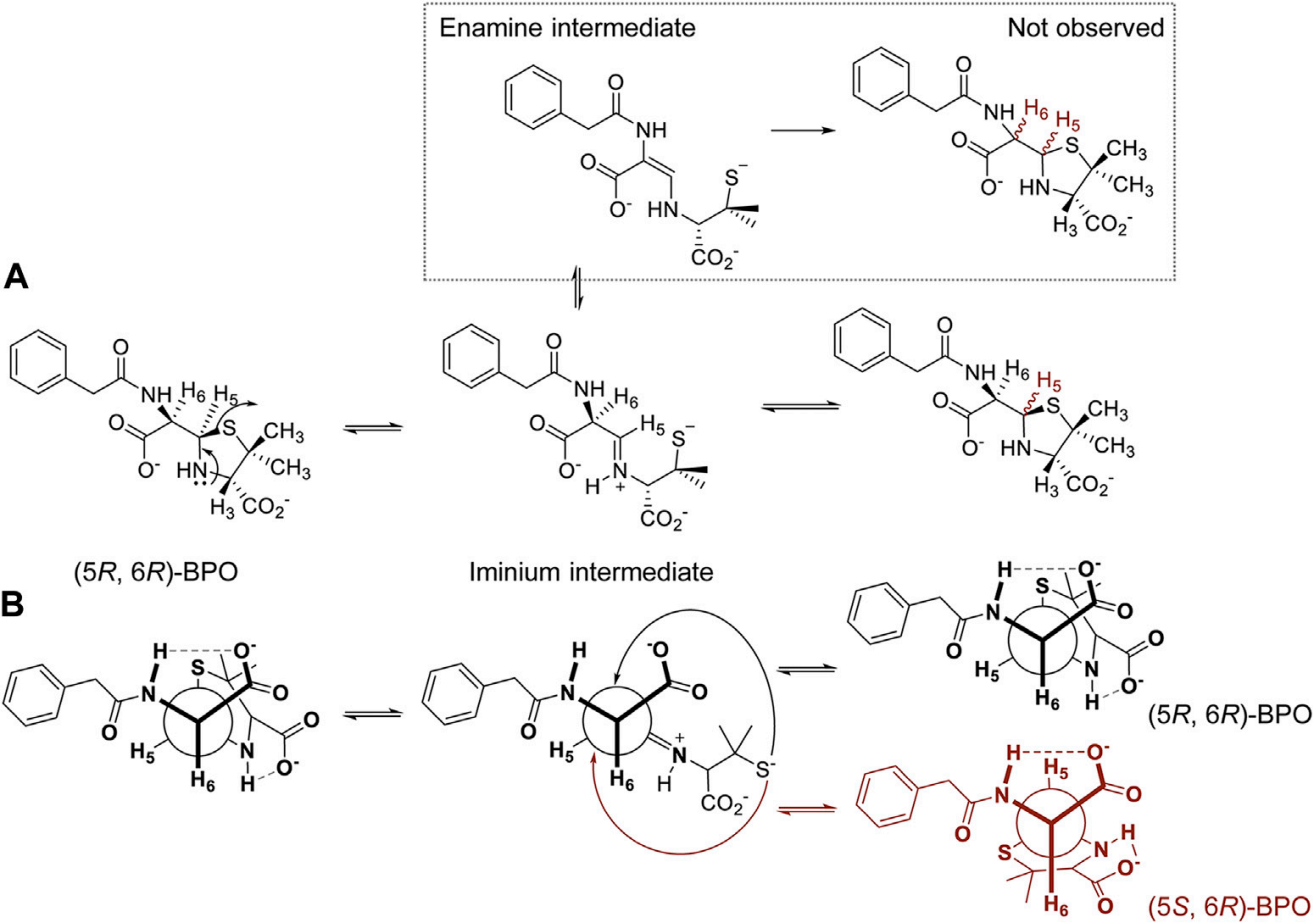
Epimerization process of (5*R*,6*R*)-BPO. **(A)** Proposed mechanism through an iminium intermediate and **(B)** same mechanism represented by the C6-C5 bond Newman projection.

In the present study, (5*R*,6*R*)-BPO initially formed resulted not completely stable in solution. While maintaining the carbon skeleton of the structure, the stereochemistry of one of the three stereocenters is altered (Ghebre-Sellassie et al., 1984; Haginaka and Wakai 1985). Although the formation of different degradation products had been previously proposed (Davis et al., 1991), epimerization rate is considerably greater and the formation of other products is almost negligible. The compound evolved reaching an equilibrium in which 70% of the (5*S*,6*R*)-BPO epimer is formed. The mechanism involved has been proposed through both the formation of an emanime (Davis and Page 1985) or the formation of an iminum intermediate (Ghebre-Sellassie et al., 1984; Branch et al., 1987; Llinás et al., 2001). However, the enamine pathway has been proposed to occur at pH above 12 (Davis et al., 1991), and has not been considered in this study, since the epimerization of C-6 is not observed experimentally (Figure 5).

In 7–7.4 pH range, the formation of an iminium intermediate promotes the opening of the thiazolidine ring. This implies breaking the covalent bond between C-5 and the sulfur atom in position 1, and therefore the free rotation between C-3, C-2 and S-1. This is a reversible process in which the ring closure through the intramolecular nucleophilic attach from the sulfur atom to the iminium C-5 can occur from the two different faces of the iminium, forming thus the two diastereoisomers observed. This closing process could be expected to occur faster than the conformational change, however, as the ring closure is a 5-*endo*-*trig* process, which is assumed to be unfavorable (Baldwin 1976), it may allow sufficient time for a conformational change. This process is described to be faster at higher pH, being almost immediate in strong basic media (Davis et al., 1991). We also observed a strong influence of temperature, reaching the equilibrium in 48 h in PBS/D_2_O at room temperature but taking 7 days at 4°C. More than one week is necessary to reach the equilibrium when the solution is in D_2_O. Experimentally, no hydrolysis of the iminium intermediate or intramolecular reaction between C-3 carboxylate and C-5 has been observed.

PO is the current available minor determinant reagent for skin test. Its formation may involve C-5 epimerization process, since the two epimers (50:50%) can be observed in freshly prepared solutions. These are very stable compounds in aqueous solutions, as they contain 50% of the product retaining original stereochemistry of BP for the studied time (seven days).

Bu-BPO was used as model compound to evaluate the stability of the major determinats reagents for skin test. Bu-BPO resulted completely stable in aqueous solutions and PBS at room temperature. Epimerization was oserved when increasing basic conditions to pH = 10.2, where the C-5 epimerization ocurrs, but with a slower rate that in the case of BPO. Seven days were necessary to observe the formation of 55% of the (5*S*,6*R*)-Bu-BPO epimer. This is consistent with previous results described in the bibliography in which the esterification of the carboxy group reduced considerably the rate of spontaneous thiazolidine ring opening (Davis et al., 1991).

**FIGURE 6:**
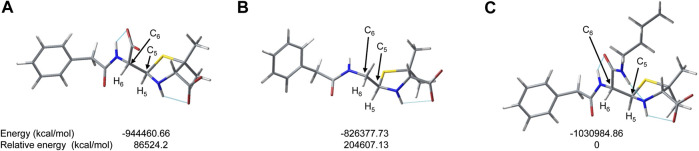
DFT Simulation at PCM(H_2_O)/B3LYP/6-311++(2 days,*p*)//PCM(H_2_O)/B3LYP/6-311 (2 days,*p*) of **(A)** (5*R*,6*R*)-BPO, **(B)** (5*R*)-PO and **(C)** (5*R*,6*R*)-Bu-BPO and their relative energies.

To deepen the stability of the different compounds, DFT calculations were made. In Figure 6 the most stable conformations of (5*R*,6*R*)-BPO, (5*R*)-PO and (5*R*,6*R*)-Bu-BPO are shown.

We can observe the formation of intramolecular hydrogen bonds that could contribute to the stabilization of the molecules. In all cases, a hydrogen bond is observed between the carboxylic group in position three and the hydrogen bonded to N-4 of the thiazolidine ring (cyane lines in Figure 6). In the case of BPO, the formation of another hydrogen bond is observed between the carboxylic acid and the amide moiety bonded to C-6. This extra interaction is not possible in the case of PO, since this carboxylic group is not present in the PO structure, and this could be translated into a more efficient epimerization process. In contrast, in the case of Bu-BPO, we can also observe the formation of a third intramolecular hydrogen bond, formed between the amide moiety of the butyl substituent and the N-4 of the thiazolidine ring. In this case, the formation of an extra hydrogen bond could make the epimerization process for Bu-BPO less successful. Electronic effects can also contribute to this effect, since the formation of the third hydrogen bong involves that N-4 of the thiazolidine ring does not have its electron pair available for the iminium formation, minimizing thus the epimerization rate. Althougth the formation of the hydrogen bonds is the main difference observed between PO, BPO and Bu-BPO molecules, we can not exclude other effects when molecules are in saline solutions. It is noteworthy that the DFT calculations using a polarizable continuum model (PCM) results in a negligible relative energy difference between the epimers ((5*R*)-PO/(5*S*)-PO; (5*R*,6*R*)-BPO/(5*S*,6*R*)-BPO and (5*R*,6*R*)-Bu-BPO/(5*S*,6*R*)-Bu-BPO), see ESI), so the driving force for the epimerization process is not clear.

To gain insight into how the relative spatial arrangement of a simple carbon (C-5) in the determinant structure can affect the interaction with the immune system, we selected a determinant model that allows the *in vitro* evaluation of the product retaining original BP stereochemistry, as well as constant proportions of its epimer. Running RAST inhibition experiments requires the inhibitors to be in neutral pH aqueous solution for 18 h. Since BPO is not stable at this conditions, due to epimerization; and PO is already supplied as mixture of epimers (50:50), Bu-BPO was selected as model determinant that allows evaluating both epimers as controling the content of epimers is possible. Therefore, we studied the correlation between Bu-BPO chemical structures and their recognition by sIgE from patients allergic to BP. From the analysis of data, we have observed that, as a general trend, sIgE recognizes Bu-BPO determinants with a lower degree of recognition with increasing proportion content of the C-5 epimer. The way in which the stereochemistry of C-5 of Bu-BPO affects IgE recognition is independent on the patient and the sIgE levels. This higher IgE specificity to (5*R*,6*R*)-Bu-BPO, which preserves the initial stereochemistry of BP, is better visualized with IC50 values. Significant lower concentrations to get the same 50% of inhibition were needed for the inhibitor one compared with the inhibitors two and three to get the same 50% of inhibition. These findings indicate, as a general rule, that both epimers of Bu-BPO determinants are specifically recognized by sera from BP-allergic patients, although the tridimensional conformation of C-5 seems to refine the extent of recognition in a high percentage of cases.

These stability and sIgE recognition data could be somewhat extrapolated about their implications into current skin reagents of BP, and their diagnostic implications, as follows: 1) the major determinant, BP-OL, is stable enough in solution to perform the *in vivo* assay, as no epimerization of this benzylpencilloyl amide form (Bu-BPO) occurs at neutral pH; 2) the minor determinant, PO, includes both epimers at 50:50 proportion, which is stable and constant to perform the *in vivo* assay; 3) regarding the BPO minor determinant ((5*R*,6*R*)-BPO (acid)), although it would contain higher amount of the one with sterochemistry better recognized *in vitro* (inhibitor 1), since it epimerizes in neutral pH solution, the reagent present in the skin test would always be a mixture of compounds in different proportions, impairing its sensitivity and impeding its reproducibility.

## Conclusion

The chemical stability of BP skin test reagents has influenced the evolution of the composition of the commercial available kits available, as this requirement is necessary for improving the quality and standardization of the product. Althought the epimerization of (5*R*,6*R*)-BPO at C-5 position in aqueous solutions is a well-known process, we provide a detailed study of the chemical stability of BP determinants at pH conditions normally used in order to further understand this progress. Our findings indicate that the epimerization rate is influenced by the structure of the determinant, changing dramatically also the kinetics of the process. *In vitro* immunoassays results show the importance of the spatial configuration of C-5 of benzylpenicilloyl determinants in the IgE recognition, with the original (5*R*,6*R*)-configuration as the best recognized. Any change, albeit small, in the chemical structure of the antigenic determinant of BP significantly affects IgE recognition. Therefore, the inclusion of stereochemically pure compounds or mixtures may have important implications for the sensitivity of both *in vivo* and *in vitro* diagnostic tests.

The conclusions drawn for the BP determinants in this study could serve as basis for the evaluation of the determinants derived from the rest of penicillins. In addition, the conditions of pH and temperature in which these reagents can be handled, for avoiding degradation or epimerization, is crucial to properly use standardized reagents that lead to reproducible results.

## Data Availability

The raw data supporting the conclusion of this article will be made available by the authors, without undue reservation, to any qualified researcher.
