# Supplementary material for: The Role of Benzylpenicilloyl Epimers in Specific IgE Recognition
Source: Front Pharmacol. 2021 Feb 26;12:585890. doi: 10.3389/fphar.2021.585890 (PMC7952312; doi:10.3389/fphar.2021.585890)
Supplement: Supplementary file 1 [file datasheet1.docx]

The Role of Benzylpenicilloyl Epimers in Specific IgE Recognition

Cristobalina Mayorga^1,2,3^, Maria I. Montañez^1,3*^, Francisco Nájera^3,4^, Gador Bogas^1,2^, David Rodríguez Gil^5^, Ricardo Palacios^5^, Maria J. Torres^1,2,3,6^, Yolanda Vida^3,4*^, Ezequiel Perez-Inestrosa^3,4*^

^1^Allergy Research Group, Instituto de Investigación Biomédica de Málaga-IBIMA, 29009, Málaga, Spain

^2^Allergy Unit, Hospital Regional Universitario de Málaga, 29009, Málaga, Spain

^3^Centro Andaluz de Nanomedicina y Biotecnología-BIONAND. Parque Tecnológico de Andalucía, C/ Severo Ochoa, 35, 29590 Campanillas, Málaga, Spain

^4^Universidad de Málaga-IBIMA, Dpto. Química Orgánica, Campus de Teatinos s/n, 29071 Málaga, Spain

^5^Diater Laboratorios S.A., Leganés, Madrid. Spain.

^6^Universidad de Málaga-IBIMA, Dpto. Medicina, Campus de Teatinos s/n, 29071 Málaga, Spain

**Table of contents**

Clinical characteristics of diagnosed patients included in the study 2

NMR Studies 3

Computational Studies 8

References 10

**Clinical characteristics of diagnosed patients included in the study**

**Table S1.** Clinical characteristics of patients diagnosed with an immediate allergic reaction to BP included in the study.

| **Case** | **Age** | **Sex** | **Reaction** | **Intradermal ST (mm)** | **RAST (%)** |
| --- | --- | --- | --- | --- | --- |
| 1 | 78 | M | Anaphylaxis | +PPL (7x5), +MDM (5x3) | **38.3** |
| 2 | 39 | M | Anaphylaxis | +PPL (5x5), +MDM (5x4) | **35.7** |
| 3 | 70 | F | Anaphylaxis | +BP-OL (5x4), +PO (5x4) | **21.1** |
| 4 | 54 | F | Urticaria | +BP-OL (5x5), +PO (5x5) | **13.5** |
| 5 | 55 | M | Anaphylaxis | +PPL (7x5), +MDM (5x4) | **15.1** |
| 6 | 31 | M | Anaphylaxis | +PPL (5x4), +MDM (5x3) | **13.3** |
| 7 | 34 | M | Urticaria | +PPL (5x5), +MDM (6x3) | **11.9** |
| 8 | 38 | F | Anaphylaxis | +PPL (6x5) | **20.0** |
| 9 | 34 | M | Urticaria | +PPL (5x5), MDM (5x4) | **7.5** |
| 10 | 54 | F | Urticaria | +BP-OL (7x5), +PO (6x5) | **9.6** |
| 11 | 70 | M | Anaphylaxis | +BP-OL (6x5), +PO (5x5) | **4.6** |

BP-OL: benzylpenicilloyl-octa-L-lysine; F: female; M: male; ST: Skin test; MDM: minor determinant mixture; PO: Penilloate; PPL: Penicilloyl-octa-lysine; RAST: RadioAllergoSorbent test

**NMR Studies**

**Figure S1.** ^1^H-NMR spectra of a solution of **BP** in D_2_O pD~6 at 23ºC, a) freshly prepared, b) after 48h and c) in PBS/D_2_O pD~7.4 at 23ºC after 7 days.

**Figure S2.** ^1^H-NMR spectra of a) **BP** and b) **BPO** in D_2_O, pD~6 at 23ºC, freshly prepared.

**Figure S3.** ^1^H-NMR spectra of a solution of **BPO** in D_2_O pD~6 at 23ºC, a) freshly prepared and after b) 15 h, c) 24 h, d) 48 h and e) 7 days.

**Figure S4.** ^1^H-NMR spectra of a solution of **PO** in PBS/D_2_O pD~7.4 at 23ºC, a) freshly prepared, b) after 48h and c) after 7 days at 4ºC.

**Figure S5.** ^1^H-NMR spectra of a solution of **PO** in D_2_O pD~6 at 23ºC, a) freshly prepared, b) after 48h.

**Figure S6.** ^1^H-NMR spectra of a solution of **Bu-BPO** in D_2_O, pD~6, at 23ºC, a) freshly prepared and after b) 7 days.

**Figure S7.** ^1^H-NMR spectra of a solution of **Bu-BPO** in PBS/D_2_O, pD~7.4, at 23ºC, a) freshly prepared and after b) 7 days.

**Figure S8.** ^1^H-NMR spectra of a solution of **Bu-BPO** in Na_2_CO_3_ buffer, pH~10.2, at 23ºC, a) freshly prepared and b) after 48 h.

**Computational Studies**

Since the formation of hydrogen bonds could affect the epimerization process of these molecules, we decided to accomplish a potential energy scan using the dihedral angle (H_6_-C_6_-C_5_-H_5_) as variable (Table S2). Therefore, we cover all the possibilities of hydrogen bond formation implying the NH atoms in the thiazolidine moiety. In almost all geometries found, we got that is possible the formation of one or several hydrogen bonds (see Table S2). In order to obtain additional insights into the nature of these hydrogen bonds further studies have been done.

One of the most useful tools to study atomic and molecular interactions, mainly hydrogen bonds, is the topological analysis based in the Bader theory of AIM (Bader 1991). The main topological parameters for these molecules can be found in Table S3.

According to this theory, the chemical bonds are characterized by the presence of bond critical points (BCP). This theory uses the value of electron density at the bond critical point and the electron density paths as criteria for the existence of the hydrogen bond. According the AIM analysis, a hydrogen bond occurs if the electron density (*ρ*) at the bond critical point should be between 0.002 and 0.035 au and the Laplacian of electron density (∇^2^*ρ*) should be within 0.024-0.139 au (Shishkin et al. 2006). For hydrogens bond critical points (hBCP), *ρ* usually has small values and ∇^2^*ρ* > 0, both characteristic of closed-shell interactions (Mata et al. 2010). Likewise, it is also convenient to consider the energetic properties of electron density at the hBCP (Table S3). In closed-shell interactions, as hydrogen bonds, the potential electron energy density (V) has a negative value and its absolute value should be similar to the kinetic electron energy density (G). In BPO and Bu-BPO, the electron energy density (H = G + V) has low values, indicating weak hydrogen bonds and mainly of electrostatic nature (Rozas, Alkorta, and Elguero 2000). With respect to the Eigenvalues of Hessian matrix, two of them (λ_1_ and λ_2_) usually have negative values confirming that all the hBCP correspond to true hydrogen bonds. The hydrogen bonds energy can also be evaluated from the potential energy density at each (Spackman 1999). Surprisingly, after the AIM analysis the molecule PO did not show any hBCP and maybe due to this epimerize faster.

Table S2: Torsional profiles of (*5R*,*6R*)-BPO, (*5R*,*6R*)-Bu-BPO and (*5R*)-PO varying the dihedral angle H_6_-C_6_-C_5_-H_5_ and the geometries obtained for each minima after optimization of their structures at PCM(H_2_O)/B3LYP/6-311G(2d,p).

| Compound | Torsional Profiles through the angle (H_6_-C_6_-C_5_-H_5_) | | |
| --- | --- | --- | --- |
| **(*5R*,*6R*)-BPO** |  | | |
| 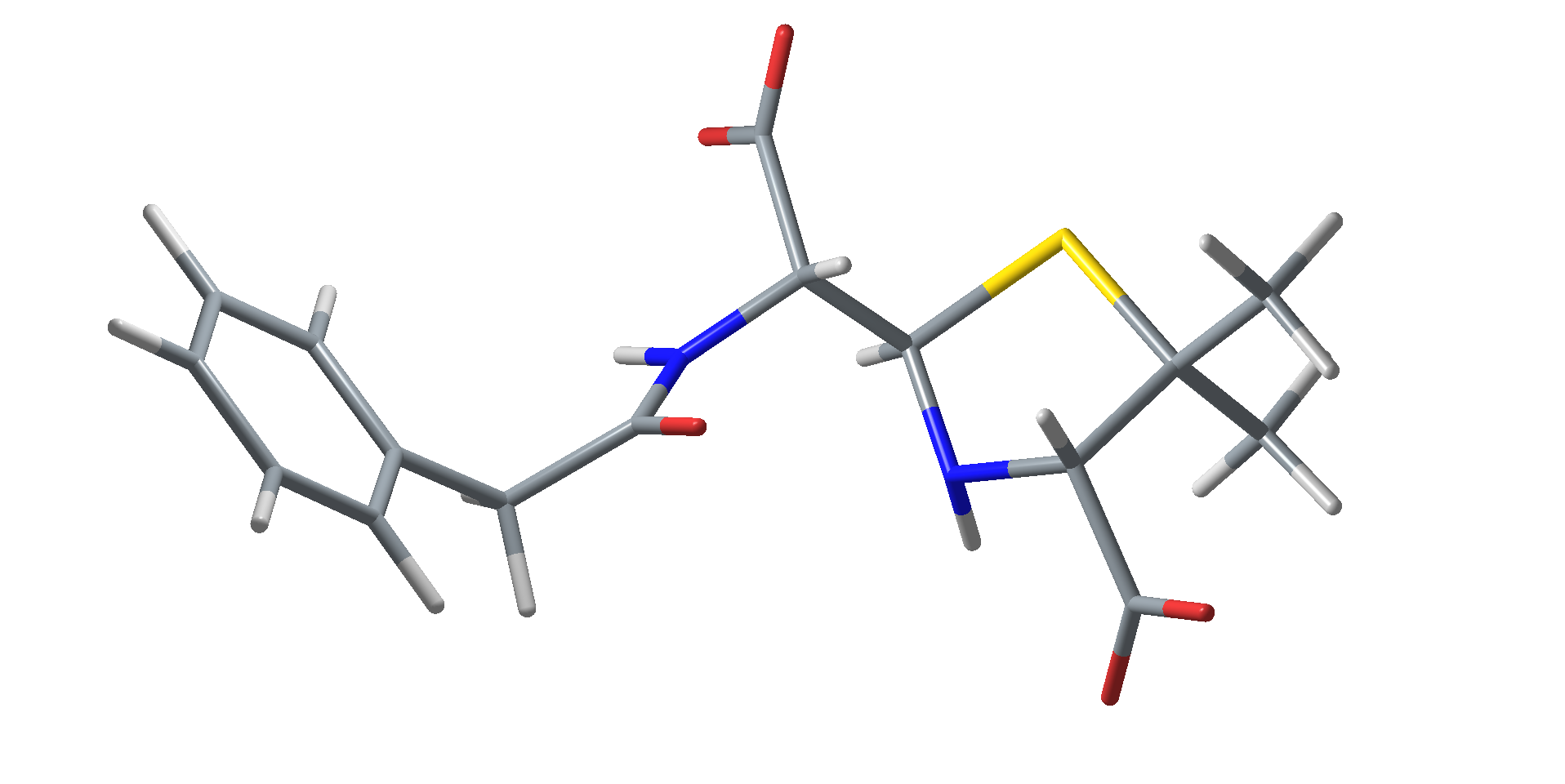 | | 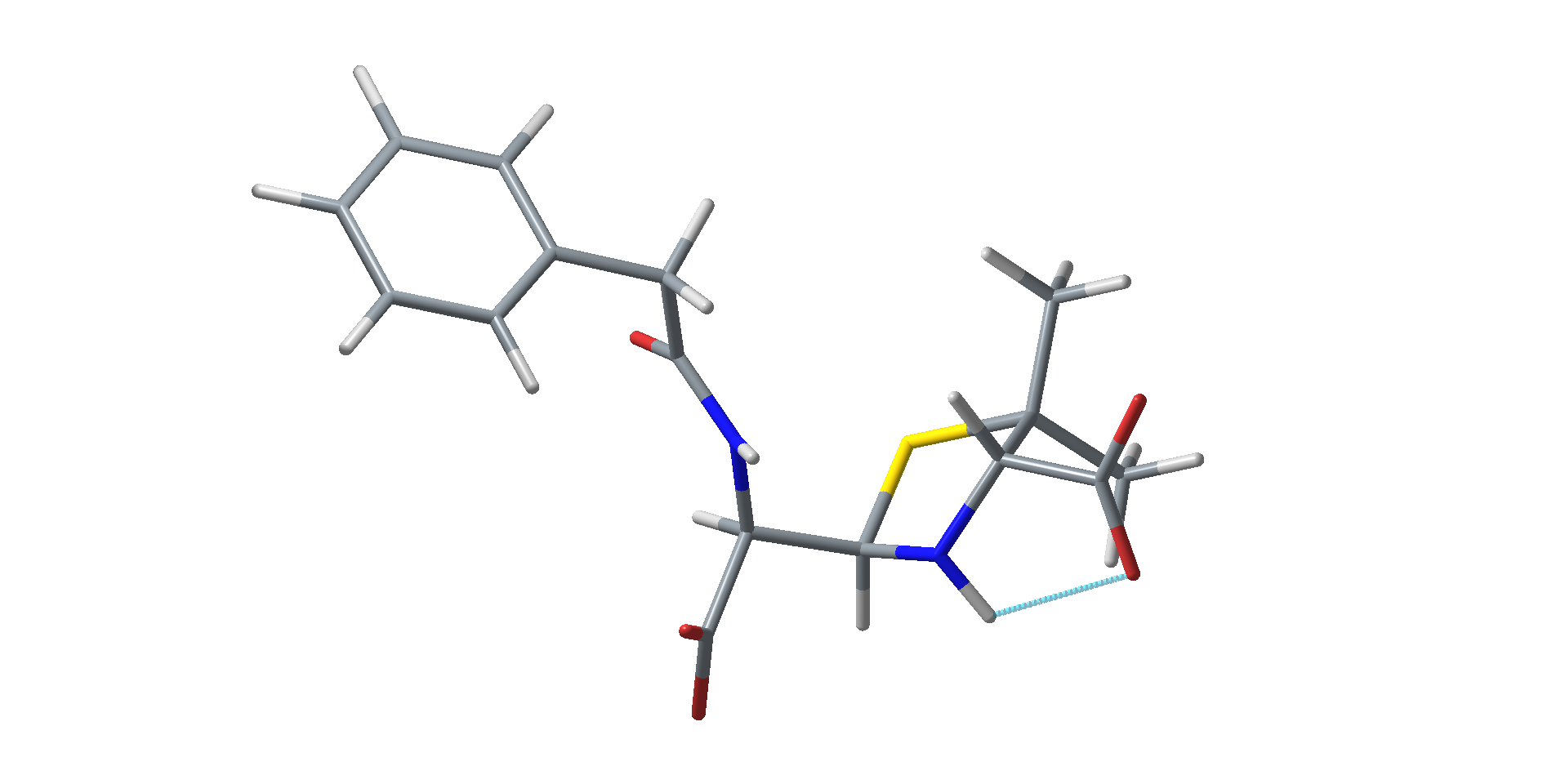 | 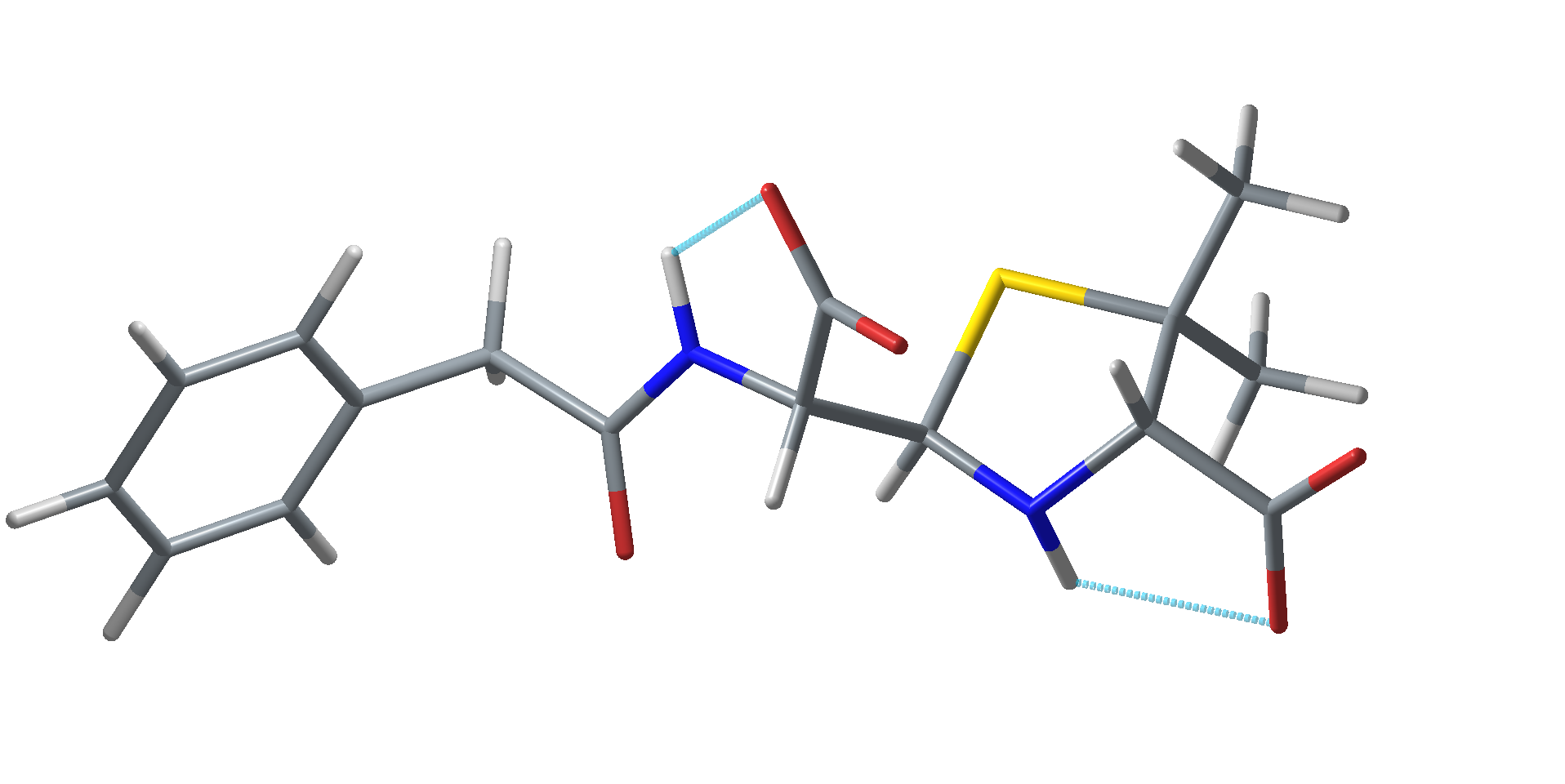 |
| Energy (kcal/mol): -944428.47 | | -944428.55 | -944429.11 |
| **(*5R*,*6R*)-Bu-BPO** |  | | |
| 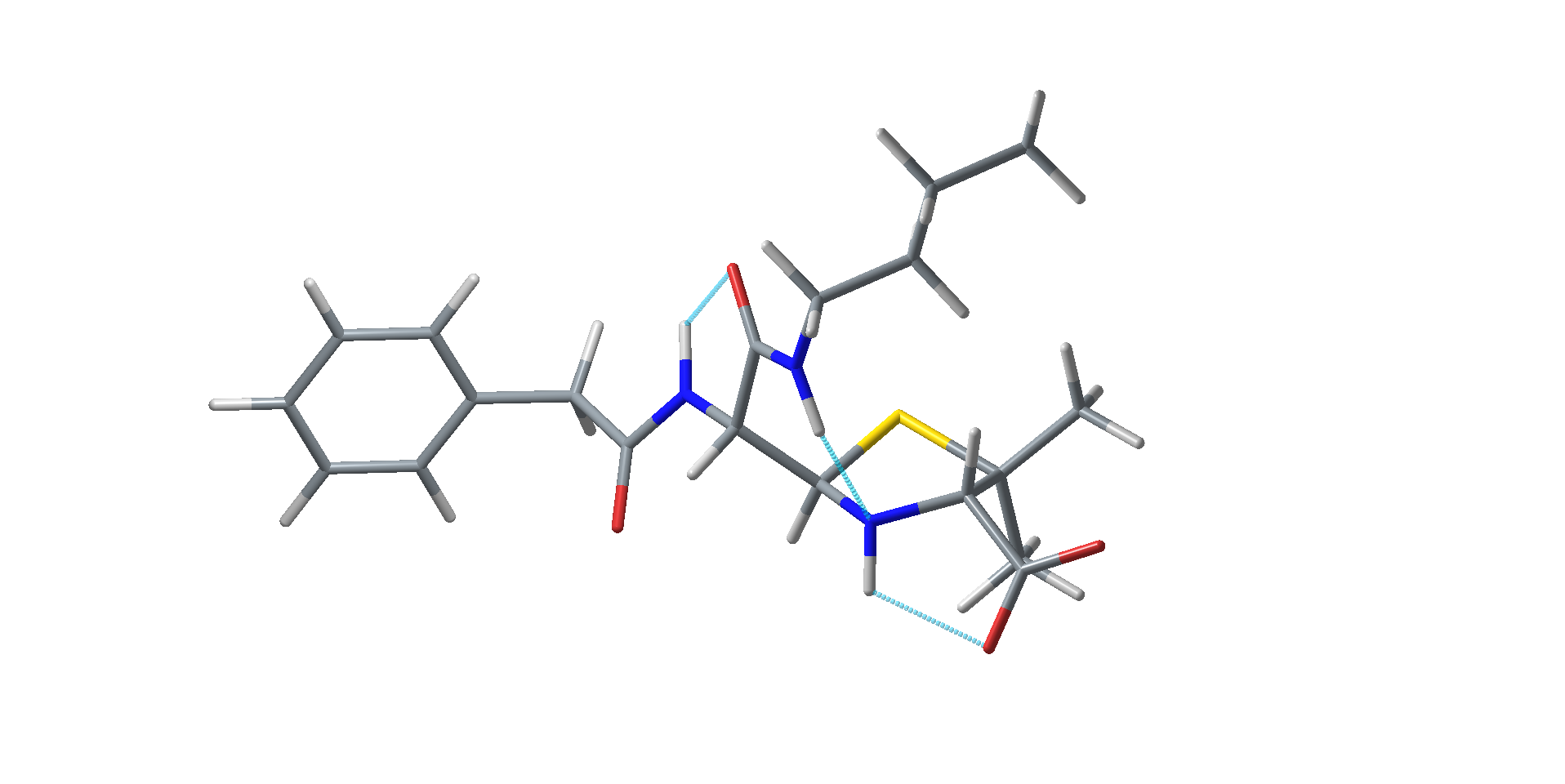 | | 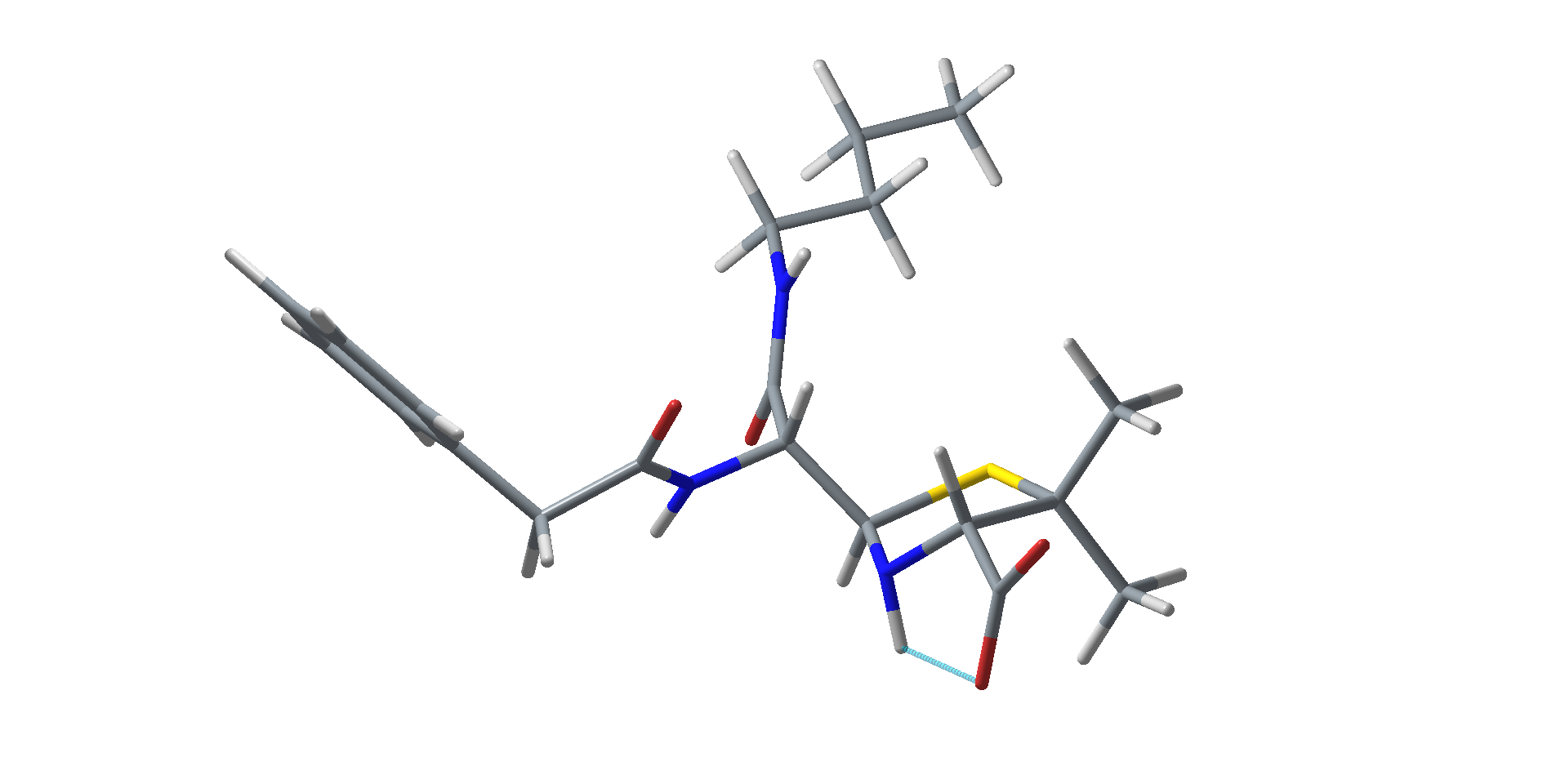 | 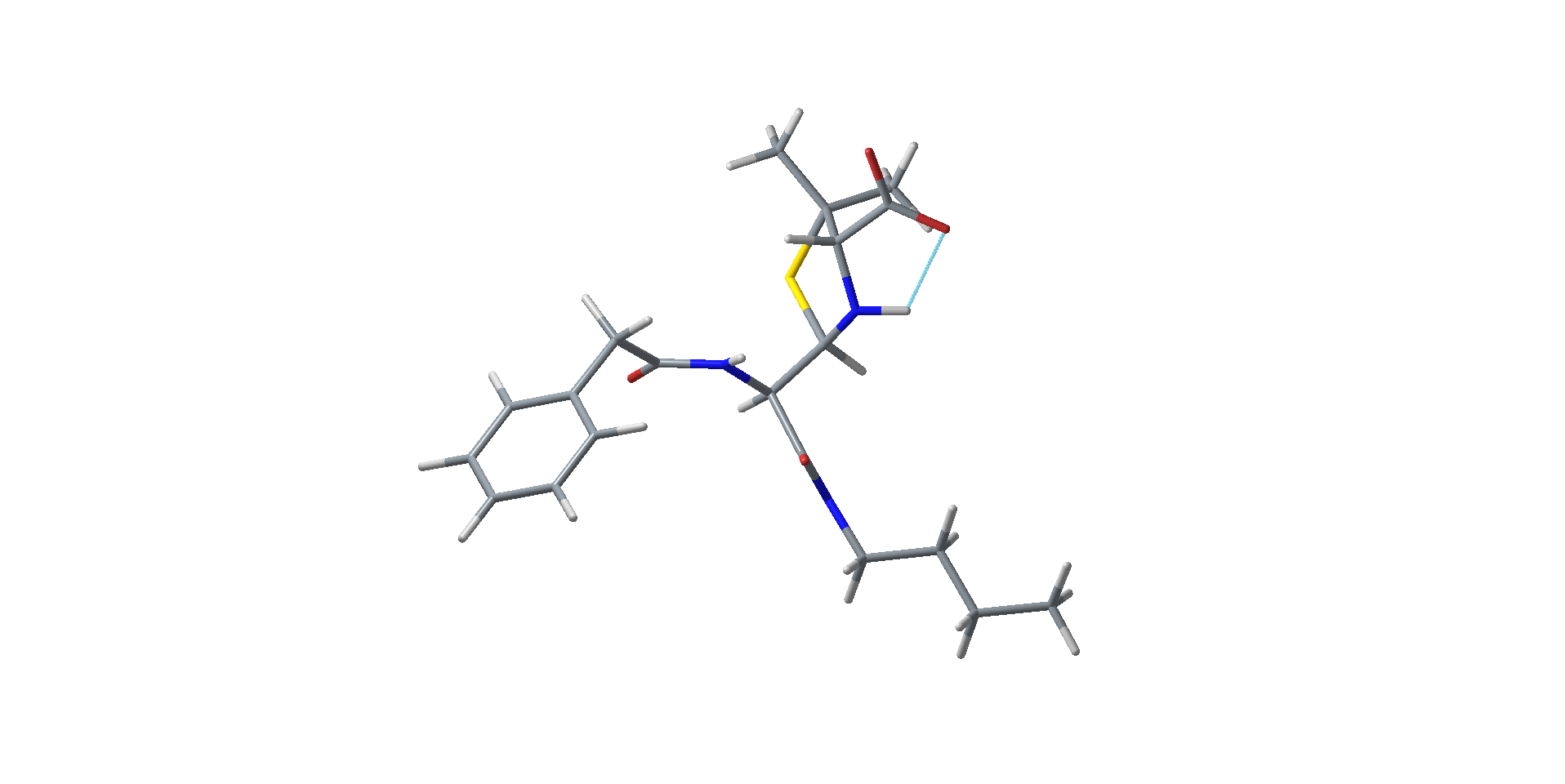 |
| Energy (kcal/mol): -1030963.04 | | -1030960.89 | -1030960.42 |
| **(*5R*)-PO** |  | | |
| 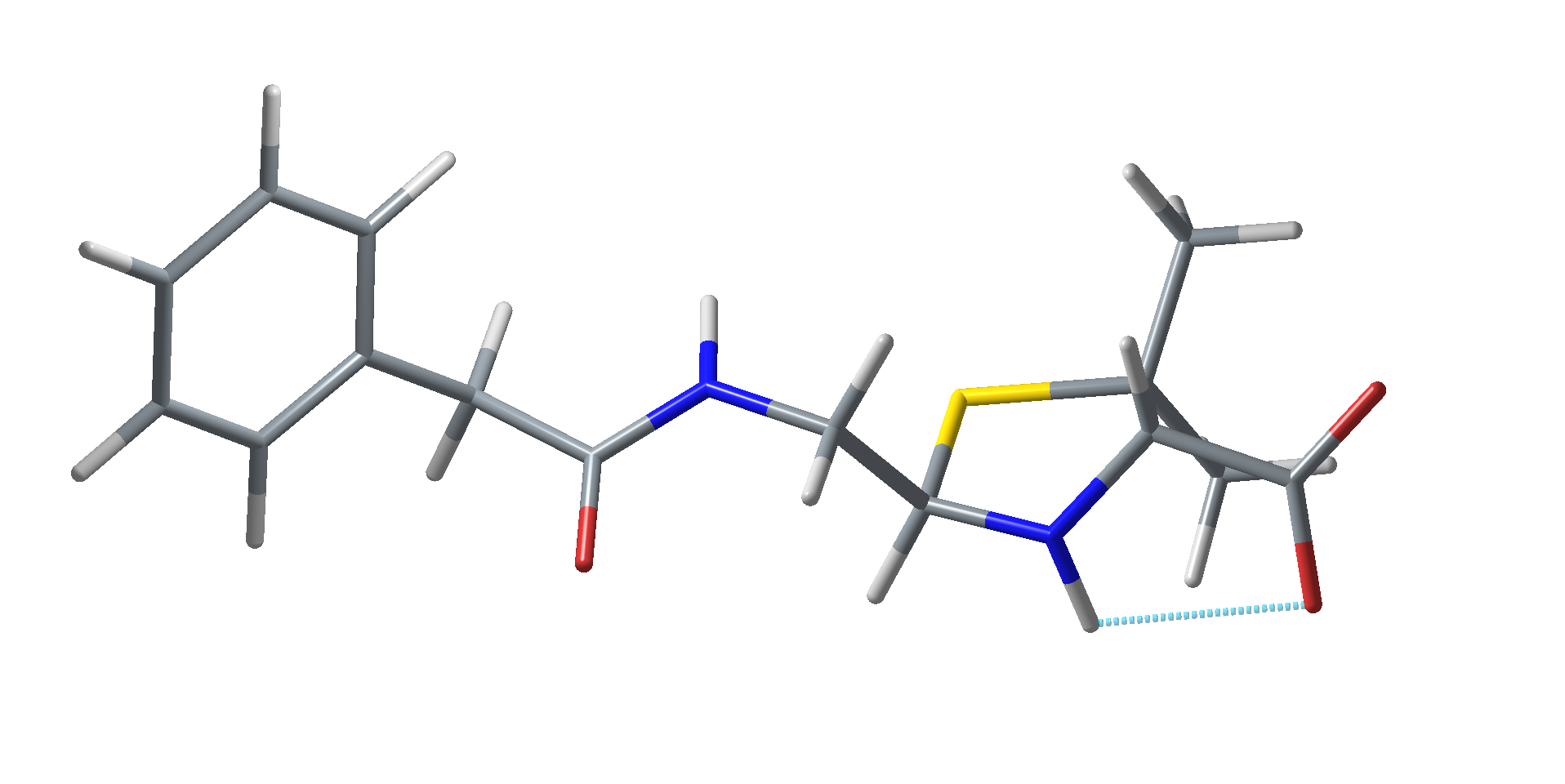 | | 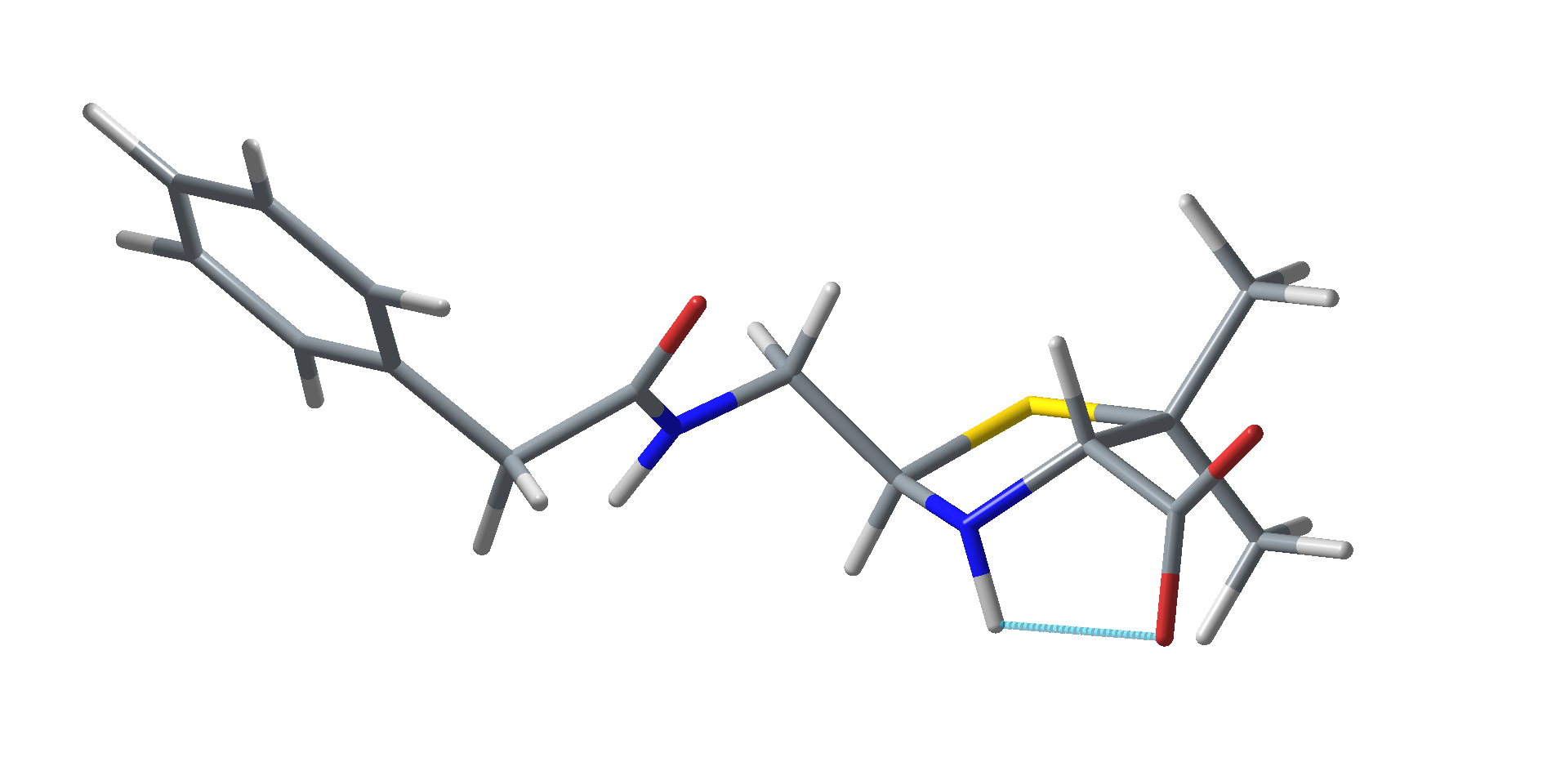 | 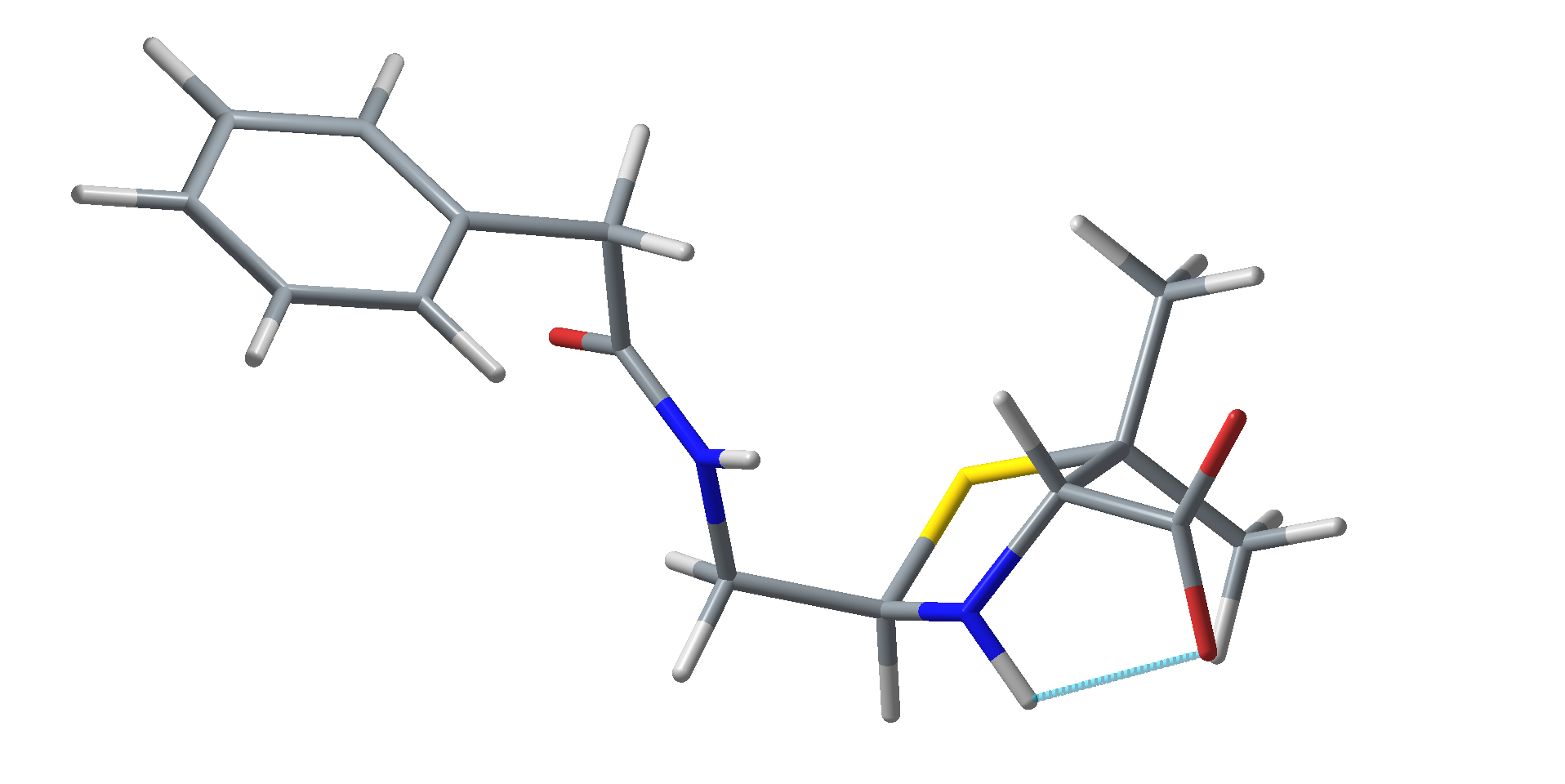 |
| Energy (kcal/mol): -826357.55 | | -826357.43 | -826356.91 |

^a^ Carbon atoms are in gray, nitrogen in blue, oxygen in red and sulfur in yellow. The hydrogen bonds founded are in cyan.

Table S3: Torsional profiles of (*5S*,*6R*)-BPO, (*5S*,*6R*)-Bu-BPO and (*5S*)-PO varying the dihedral angle H_6_-C_6_-C_5_-H_5_ and the geometries obtained for each minima after optimization of their structures at PCM(H_2_O)/B3LYP/6-311G(2d,p).

| Compound | Torsional Profiles through the angle (H_6_-C_6_-C_5_-H_5_) | | | | | | | |
| --- | --- | --- | --- | --- | --- | --- | --- | --- |
| **(*5S*,*6R*)-BPO** |  | | | | | | | |
| 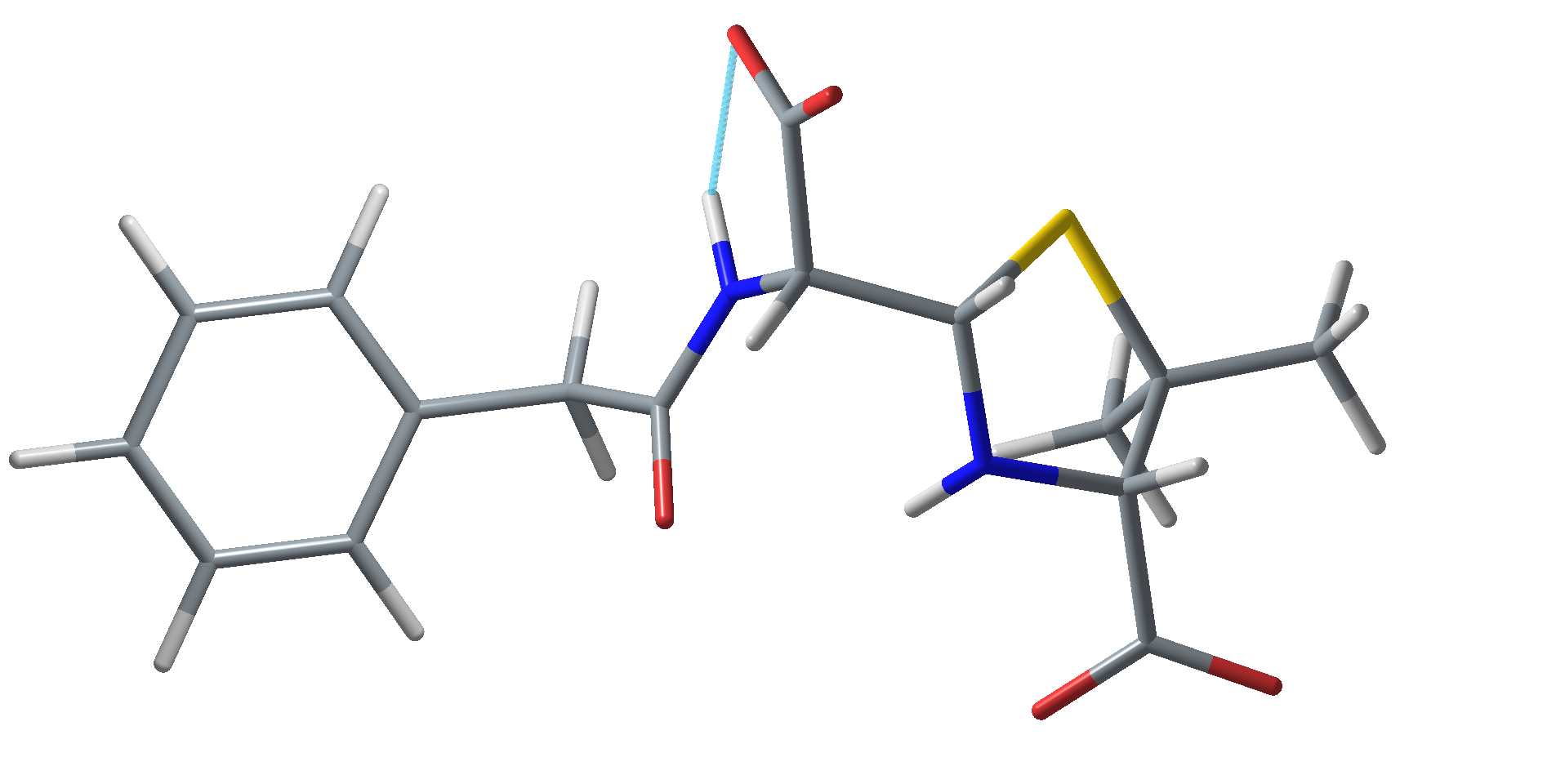 | | | 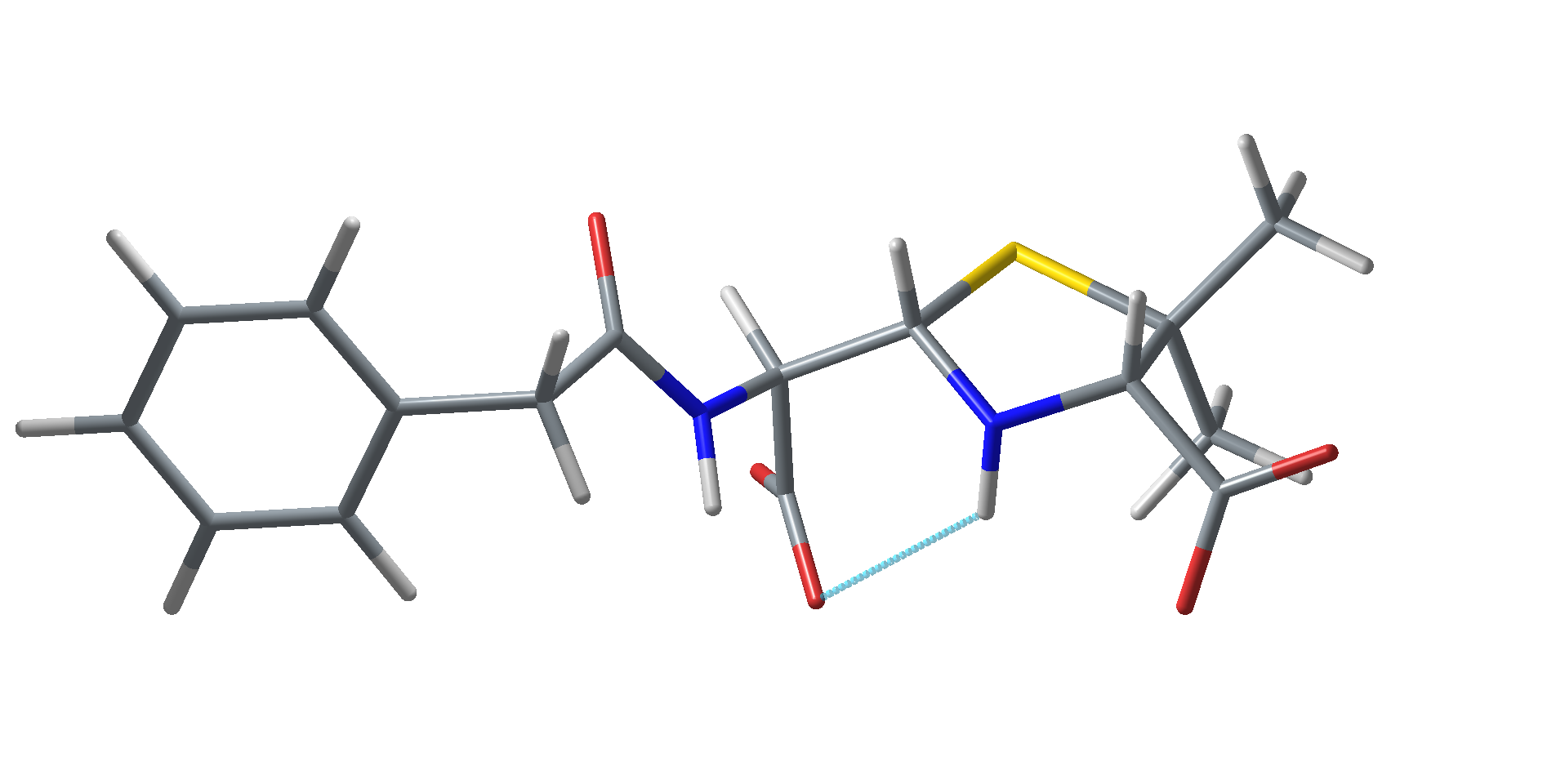 | | | 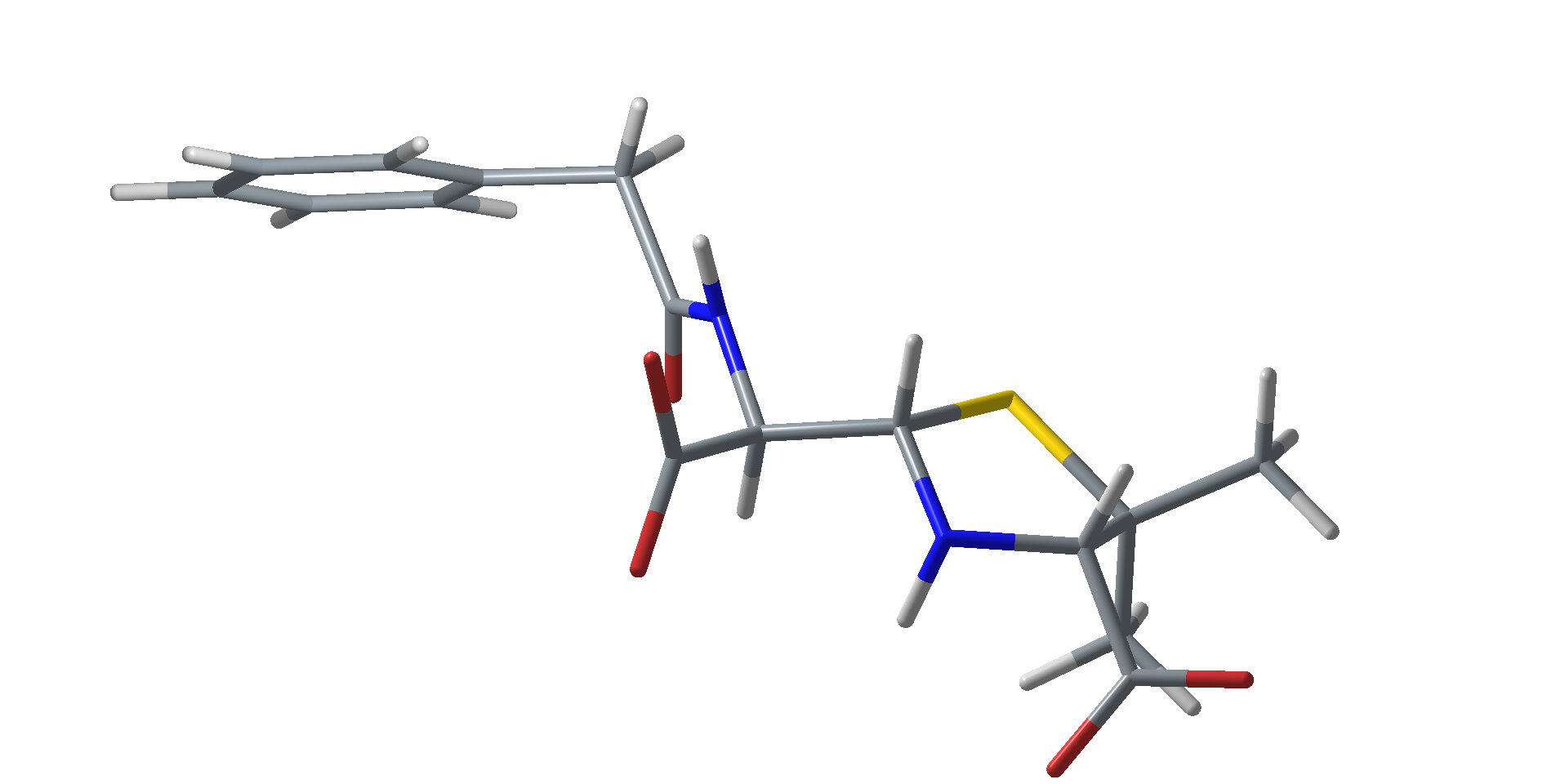 | | 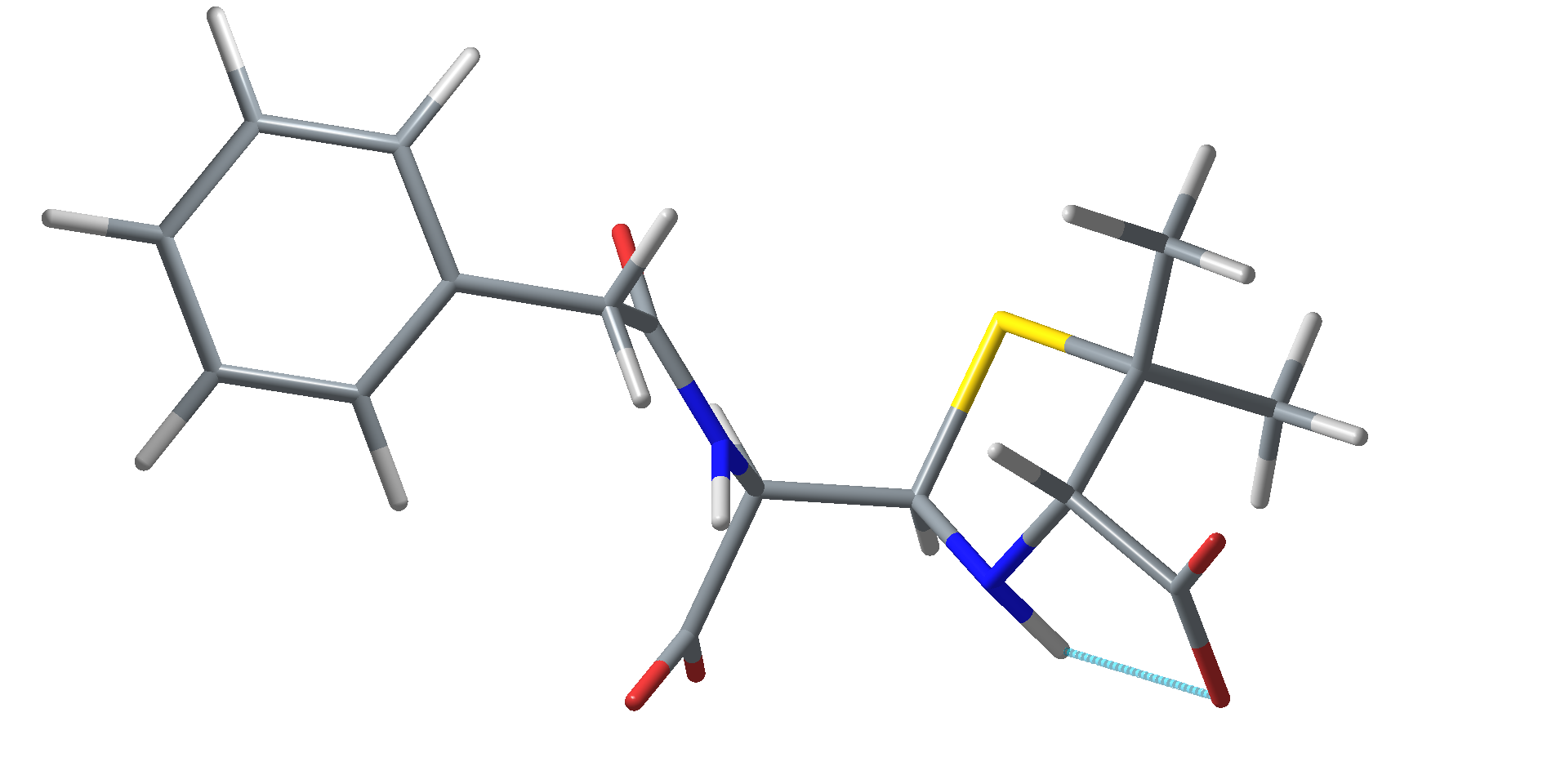 |
| Energy (kcal/mol): -944430.59 | | | -944429.37 | | | -944429.32 | | -944431.93 |
| **(*5S*,*6R*)-Bu-BPO** |  | | | | | | | |
| 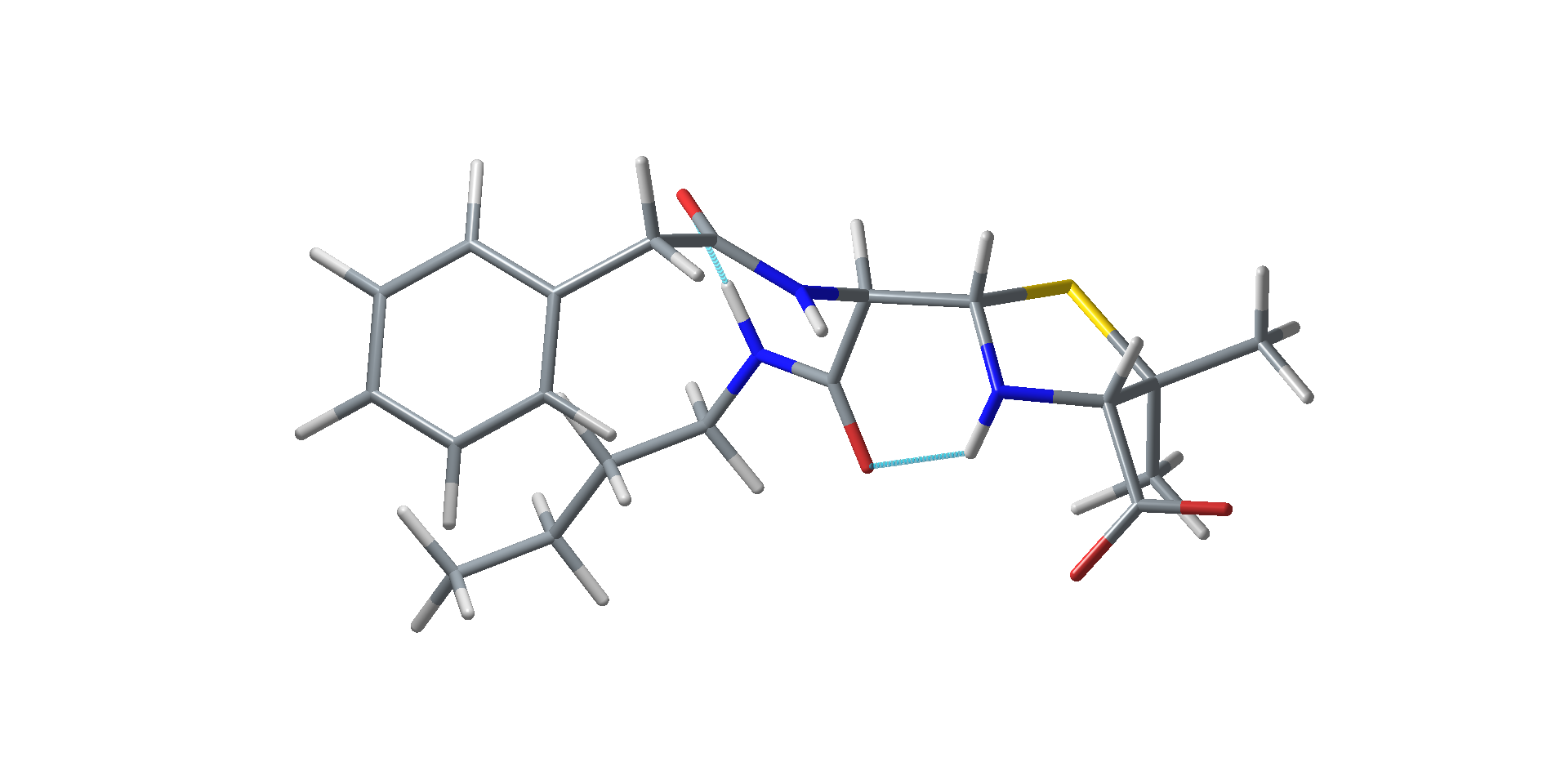 | | | | 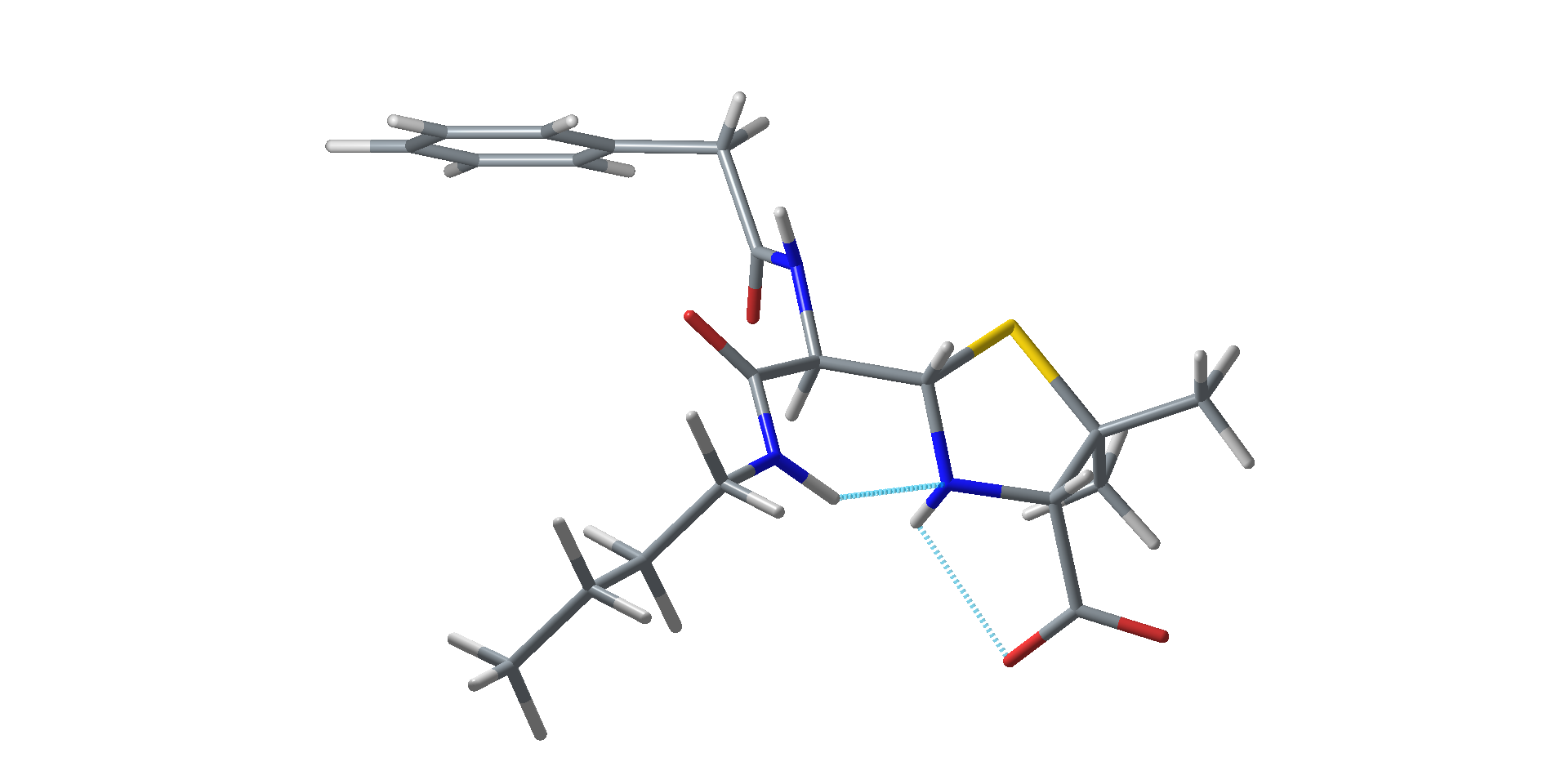 | | | 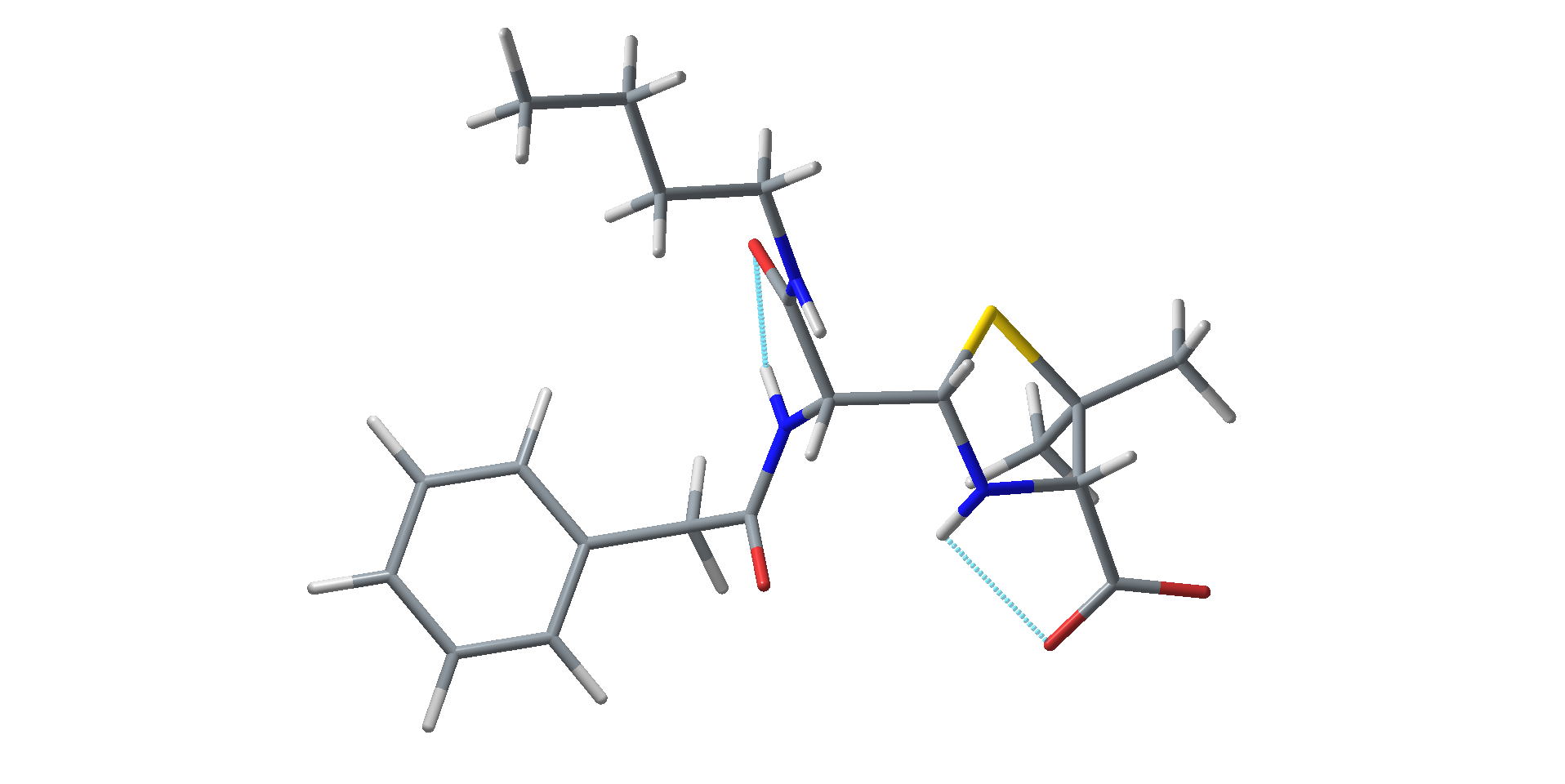 | |
| Energy: -1030961.70 kcal/mol | | | | -1030961.28 kcal/mol | | | -1030961.36 kcal/mol | |
| **(*5S*)-PO** |  | | | | | | | |
| 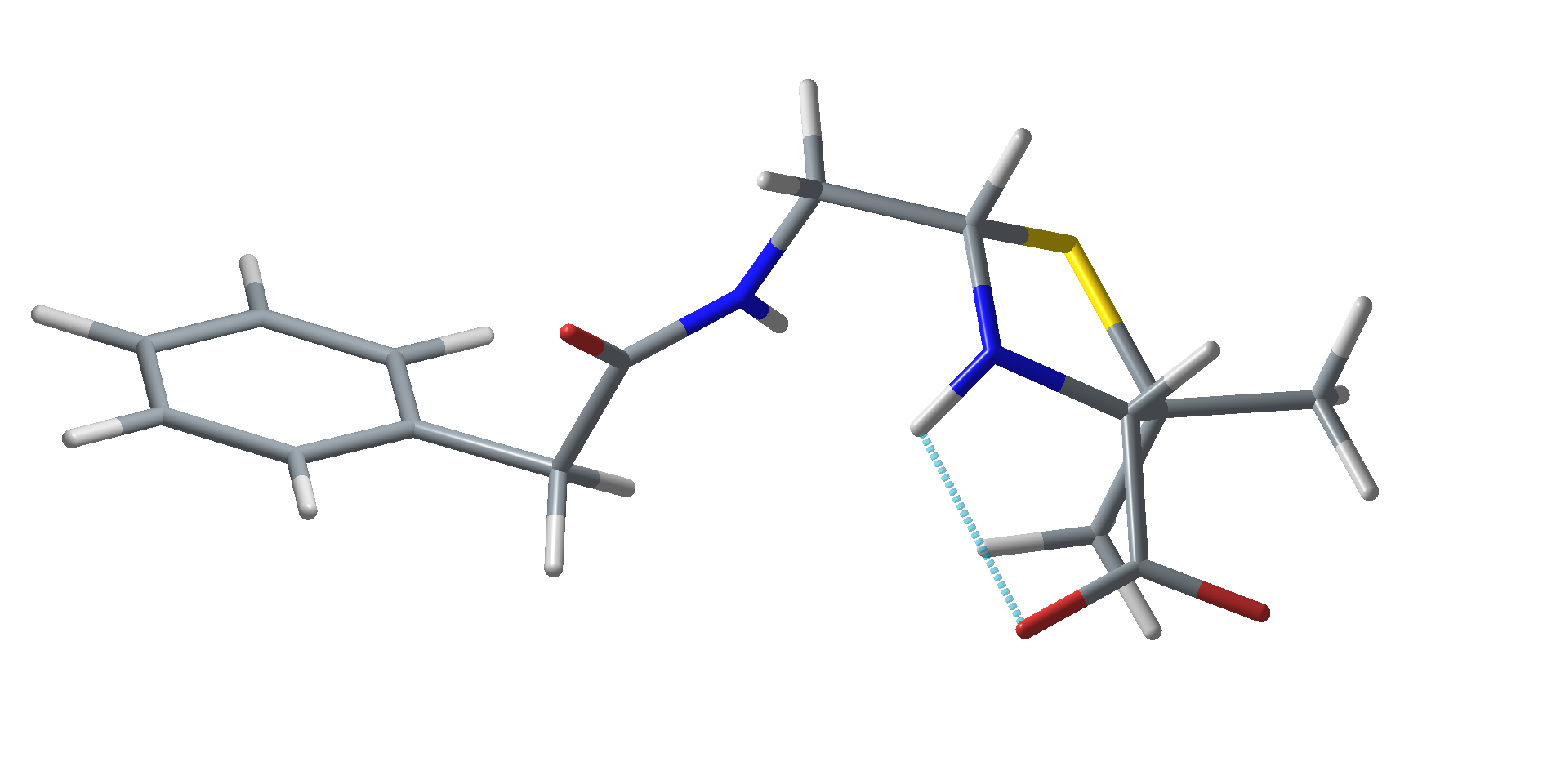 | | 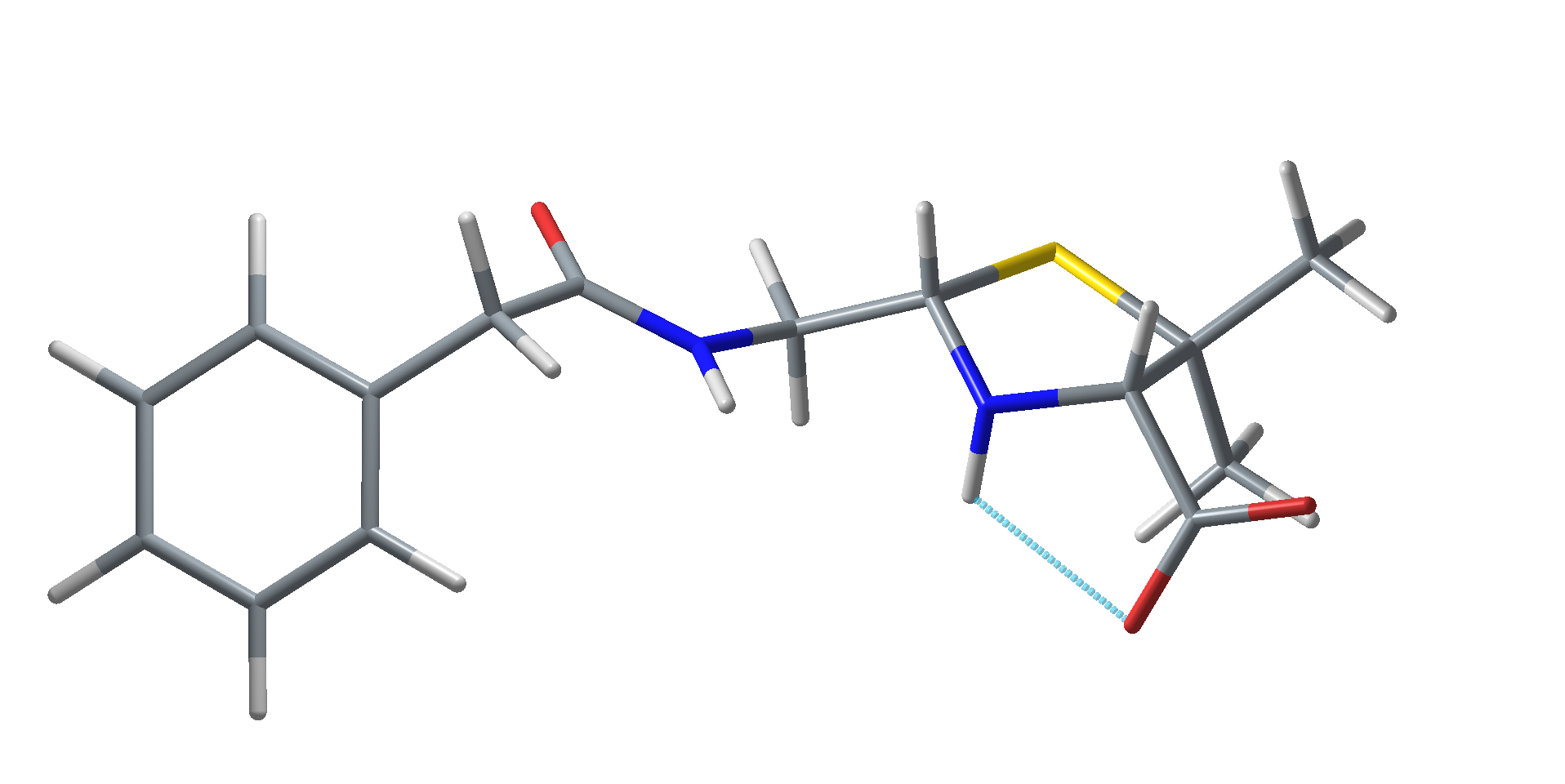 | | | 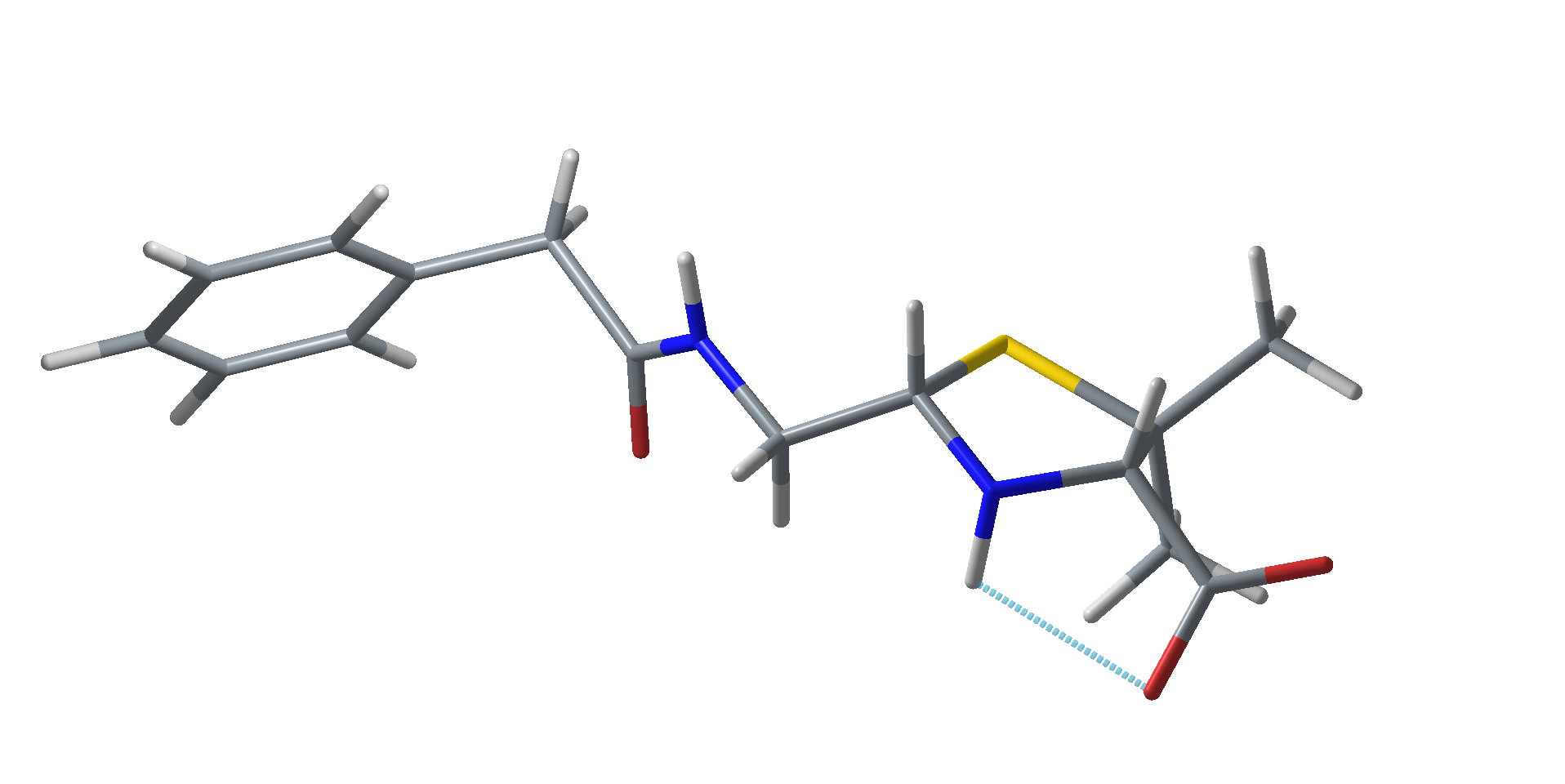 | | | 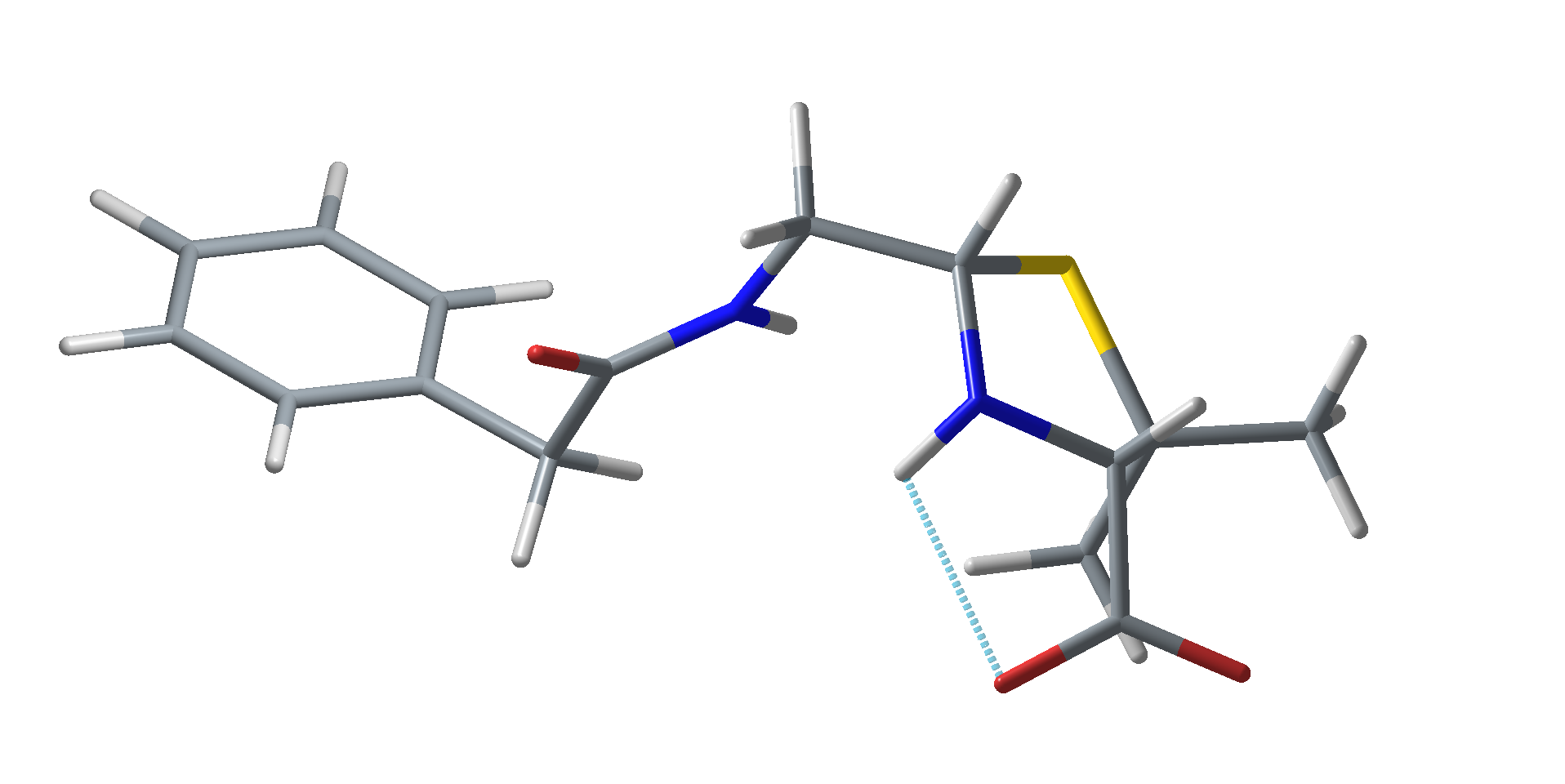 |
| Energy (kcal/mol): -826357.67 | | -826357.62 | | | -826356.60 | | | -826357.44 |

^a^ Carbon atoms are in gray, nitrogen in blue, oxygen in red and sulfur in yellow. The hydrogen bonds founded are in cyan.

Table S4: Topological parameters from the AIM analysis for BPO, Bu-BPO and PO. The Bond Critical Points (BCP) are represented in orange and the path connecting the (3,-3) and (3,-1) CP are in yellow.

| **(5*R*,6*R*)-BPO** | | | **(5*R*,6*R*)-Bu-BPO** | | **(5*R*)-PO** | | |
| --- | --- | --- | --- | --- | --- | --- | --- |
| 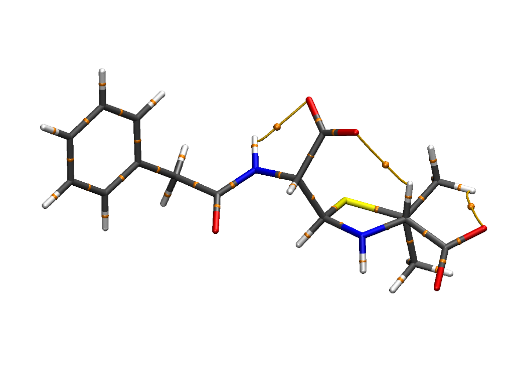 hBCP 85 | | | 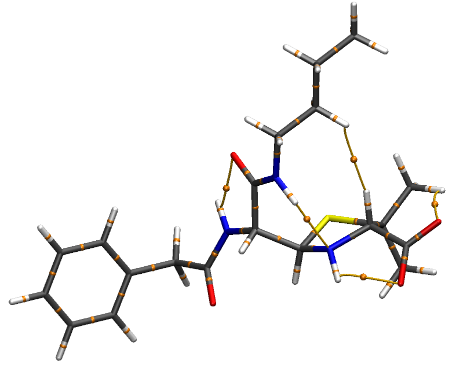 hBCP 70  hBCP 94  hBCP 102 | | 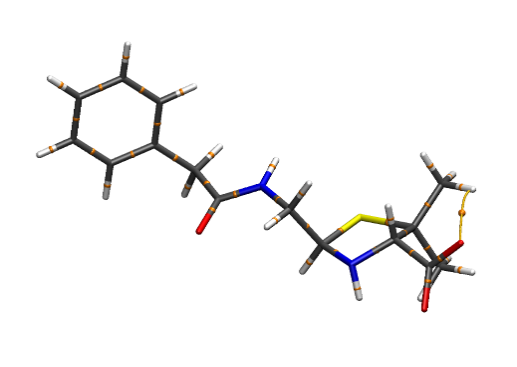 | | |
| hBCP 85  CONH···OCO | hBCP 70  R_2_NH···OCO | hBCP 94  CONH···NHR_2_ | | hBCP 102  CONH···OCNH | |  |  |
| 0.0254 | 0.0229 | 0.0223 | | 0.0224 | | *ρ*^hBCP^ | Hydrogen Bond Critical Point (hBCP)^a^ |
| 0.1058 | 0.0946 | 0.0705 | | 0.1014 | | ∇^2^*ρ*^hBCP^ |  |
| 0.0232 | 0.0208 | 0.0155 | | 0.0218 | | G | Energetic properties of electron density at hBCP^b^ |
| -0.0200 | -0.0180 | -0.0134 | | -0.0183 | | V |  |
| 0.0032 | 0.0028 | 0.0021 | | 0.0035 | | H |  |
| 0.8621 | 0.8654 | 0.8645 | | 0.8395 | | \| V \|/G |  |
| -0.0286 | -0.0242 | -0.0256 | | -0.0238 | | λ_1_ | Eigenvalues of the Hessian matrix^c^ |
| -0.0187 | -0.0138 | -0.0238 | | -0.0093 | | λ_2_ |  |
| 0.1531 | 0.1326 | 0.1199 | | 0.1344 | | λ_3_ |  |
| 0.3089 | 0.2866 | 0.4120 | | 0.2463 | | \| (λ_1_+λ_2_) \|/λ_3_ |  |
| -6.28 | -5.65 | -4.21 | | -5.74 | | E_HB_ | Hydrogen Bond Energy^d^ |
| ^a^ *ρ*: Electron density in *e/a_o_^3^* and ∇^2^*ρ*: Laplacian of electron density in *e/a_o_^5^*; ^b^ G: Lagrangian kinetic energy in *e/a_o_^3^*; V: Potential energy density in *e/a_o_^3^*; H: Energy density in *e/a_o_^3^*; ^c^ λ_1_, λ_2_, λ_3_: Eigenvalues of Hessian matrix in *au*; ^d^ E_HB_: Hydrogen Bond Energy in *kcal/mol*. | | | | | | | |

**References**

Bader, Richard F.W. 1991. “A Quantum Theory of Molecular Structure and Its Applications.” *Chemical Reviews* 91 (5): 893–928. https://doi.org/10.1021/cr00005a013.

Mata, Ignasi, Ibon Alkorta, Elies Molins, and Enrique Espinosa. 2010. “Universal Features of the Electron Density Distribution in Hydrogen- Bonding Regions: A Comprehensive Study Involving H···X (X=H, C, N, O, F, S, Cl, π) Interactions.” *Chemistry - A European Journal* 16 (8): 2442–52. https://doi.org/10.1002/chem.200901628.

Rozas, I., I. Alkorta, and J. Elguero. 2000. “Behavior of Ylides Containing N, O, and C Atoms as Hydrogen Bond Acceptors.” *Journal of the American Chemical Society* 122 (45): 11154–61. https://doi.org/10.1021/ja0017864.

Shishkin, Oleg V., Gennady V. Palamarchuk, Leonid Gorb, and Jerzy Leszczynski. 2006. “Intramolecular Hydrogen Bonds in Canonical 2‘-Deoxyribonucleotides: An Atoms in Molecules Study.” *J. Phys. Chem. B* 110 (9): 4413–22. https://doi.org/10.1021/JP056902+.

Spackman, Mark A. 1999. “Hydrogen Bond Energetics from Topological Analysis of Experimental Electron Densities: Recognising the Importance of the Promolecule.” *Chemical Physics Letters* 301 (5–6): 425–29. https://doi.org/10.1016/S0009-2614(99)00071-8.
